# Supplementary material for: New Multifunctional Agents for Potential Alzheimer’s Disease Treatment Based on Tacrine Conjugates with 2-Arylhydrazinylidene-1,3-Diketones
Source: Biomolecules. 2022 Oct 24;12(11):1551. doi: 10.3390/biom12111551 (PMC9687805; doi:10.3390/biom12111551)
Supplement: Supplementary file 1 [file biomolecules-12-01551-s001.zip › biomolecules-1956516-supplementary.pdf]

New multifunctional agents for potential Alzheimer's disease treatment based on tacrine conjugates with 2-arylhydrazilidene-1,3-diketones

Natalia A. Elkina [1], Maria V. Grishchenko [1], Evgeny V. Shchegolkov [1], Galina F. Makhaeva [2], Nadezhda V. Kovaleva [2], Elena V. Rudakova [2], Natalia P. Boltneva [2], Sofya V. Lushchekina [2,3], Tatiana Y. Astakhova [3], Eugene V. Radchenko [2,4], Vladimir A. Palyulin [2,4], Ekaterina F. Zhilina [1], Anastasiya N. Perminova [1], Luka S. Lapshin [1], Yanina V. Burgart [1], Victor I. Saloutin [1], and Rudy J. Richardson [5,6,7,8*]

[1] Postovsky Institute of Organic Synthesis, Urals Branch of Russian Academy of Sciences, Yekaterinburg, 620990, Russia; natali741258@mail.ru (N.A.E.); morozmv@gmail.com (M.V.G.); e.schegolkov@yandex.ru (E.V.S.); efzhilina@ios.uran.ru (E.F.Z.); nastena_pn@mail.ru (A.N.P.); lapshin.luka@mail.ru (L.S.L.); ya.burgart@yandex.ru (Y.V.B.); saloutin@ios.uran.ru (V.I.S.)

[2] Institute of Physiologically Active Compounds Russian Academy of Sciences, Chernogolovka, 142432, Russia; gmakh@ipac.ac.ru (G.F.M.); kovalevanv@ipac.ac.ru (N.V.K.); rudakova@ipac.ac.ru (E.V.Ru.); boltneva@ipac.ac.ru (N.P.B.); sofya.lushchekina@gmail.com (S.V.L.)

[3] Emanuel Institute of Biochemical Physics Russian Academy of Sciences, Moscow, 119334, Russia; sofya.lushchekina@gmail.com (S.V.L.); astakhova1967.t@yandex.ru (T.Y.A.)

[4] Department of Chemistry, Lomonosov Moscow State University, Moscow, 119991, Russia; genie@qsar.chem.msu.ru (E.V.R.); vap@qsar.chem.msu.ru (V.A.P.)

[5] Department of Environmental Health Sciences, University of Michigan, Ann Arbor, MI 48109 USA

[6] Department of Neurology, University of Michigan, Ann Arbor, MI 48109 USA

[7] Center of Computational Medicine and Bioinformatics, University of Michigan, Ann Arbor, MI 48109 USA

[8] Michigan Institute for Computational Discovery and Engineering, University of Michigan, Ann Arbor, MI 48109 USA

* Correspondence: rjrich@umich.edu; Tel.: +1-734-936-0769

Table of contents

Figure S1. Absorption spectra of compound **6a** (40µM), Cu^{2+} ions solution (40µM), a sum of **6a** and Cu^{2+}, their mixture, and the shift of the spectra caused by the formation of a complex. 3

Figure S2. Absorption spectra of compound **6a** (40µM), Zn^{2+} ions solution (40µM), a sum of **6a** and Zn^{2+}, their mixture, and the shift of the spectra caused by the formation of a complex. 4

Figure S3. Absorption spectra of compound **6a** (40µM), Fe^{2+} ions solution (40µM), a sum of **6a** and Fe^{2+}, their mixture, and the shift of the spectra caused by the formation of a complex. 5

Figure S4. Absorption spectra of compound **7a** (40µM), Cu^{2+} ions solution (40µM), a sum of **7a** and Cu^{2+}, their mixture, and the shift of the spectra caused by the formation of a complex. 6

Figure S5. Absorption spectra of compound **7a** (40µM), Zn^{2+} ions solution (40µM), a sum of **7a** and Zn^{2+}, their mixture, and the shift of the spectra caused by the formation of a complex. 7

Figure S6. Absorption spectra of compound **7a** (40µM), Fe^{2+} ions solution (40µM), a sum of **7a** and Fe^{2+}, their mixture, and the shift of the spectra caused by the formation of a complex. 8

Figure S7. Absorption spectra of compound **8a** (40µM), Cu^{2+} ions solution (40µM), a sum of **8a** and Cu^{2+}, their mixture, and the shift of the spectra caused by the formation of a complex 9

Figure S8. Absorption spectra of compound **8a** (40µM), Fe^{2+} ions solution (40µM), a sum of **8a** and Fe^{2+}, their mixture, and the shift of the spectra caused by the formation of a complex 10

Figure S9. IC50 values for AChE inhibition by compounds **6a,7a,8a** (MEAN ± SEM, n = 3) 11
Figure S10. IC50 values for BChE inhibition by compounds **6a,7a,8a** (MEAN ± SEM, n = 3)..........................12
Figure S11. ^{1}H NMR spectrum of compound **2a**..........................13
Figure S12. ^{13}C NMR spectrum of compound **2a**..........................14
Figure S13. ^{1}H NMR spectrum of compound **2b** 15
Figure S14. ^{13}C NMR spectrum of compound **2b** 16
Figure S15. ^{1}H NMR spectrum of compound **2c**..........................17
Figure S16. ^{13}C NMR spectrum of compound **2c**..........................18
Figure S17. ^{19}F NMR spectrum of compound **2c** 19
Figure S18 ^{1}H NMR spectrum of compound **6a**..........................20
Figure S19. ^{13}C NMR spectrum of compound **6a**..........................21
Figure S20. ^{1}H NMR spectrum of compound **6b** 22
Figure S21. ^{13}C NMR spectrum of compound **6b** 23
Figure S22. ^{1}H NMR spectrum of compound **6c**..........................24
Figure S23. ^{13}C NMR spectrum of compound **6c**..........................25
Figure S24. ^{1}H NMR spectrum of compound **7a**..........................26
Figure S25. ^{13}C NMR spectrum of compound **7a**..........................27
Figure S26. ^{1}H NMR spectrum of compound **7b** 28
Figure S27. ^{13}C NMR spectrum of compound **7b** 29
Figure S28. ^{1}H NMR spectrum of compound **7c**..........................30
Figure S29. ^{13}C NMR spectrum of compound **7c**..........................31
Figure S30. ^{1}H NMR spectrum of compound **8a**..........................32
Figure S31. ^{13}C NMR spectrum of compound **8a**..........................33
Figure S32. ^{19}F NMR spectrum of compound **8a** 34
Figure S33. ^{1}H NMR spectrum of compound **8b** 35
Figure S34. ^{13}C NMR spectrum of compound **8b** 36
Figure S35. ^{19}F NMR spectrum of compound **8b**..........................37
Figure S36. ^{1}H NMR spectrum of compound **8c**..........................38
Figure S37. ^{13}C NMR spectrum of compound **8c**..........................39
Figure S38. ^{19}F NMR spectrum of compound **8c** 40

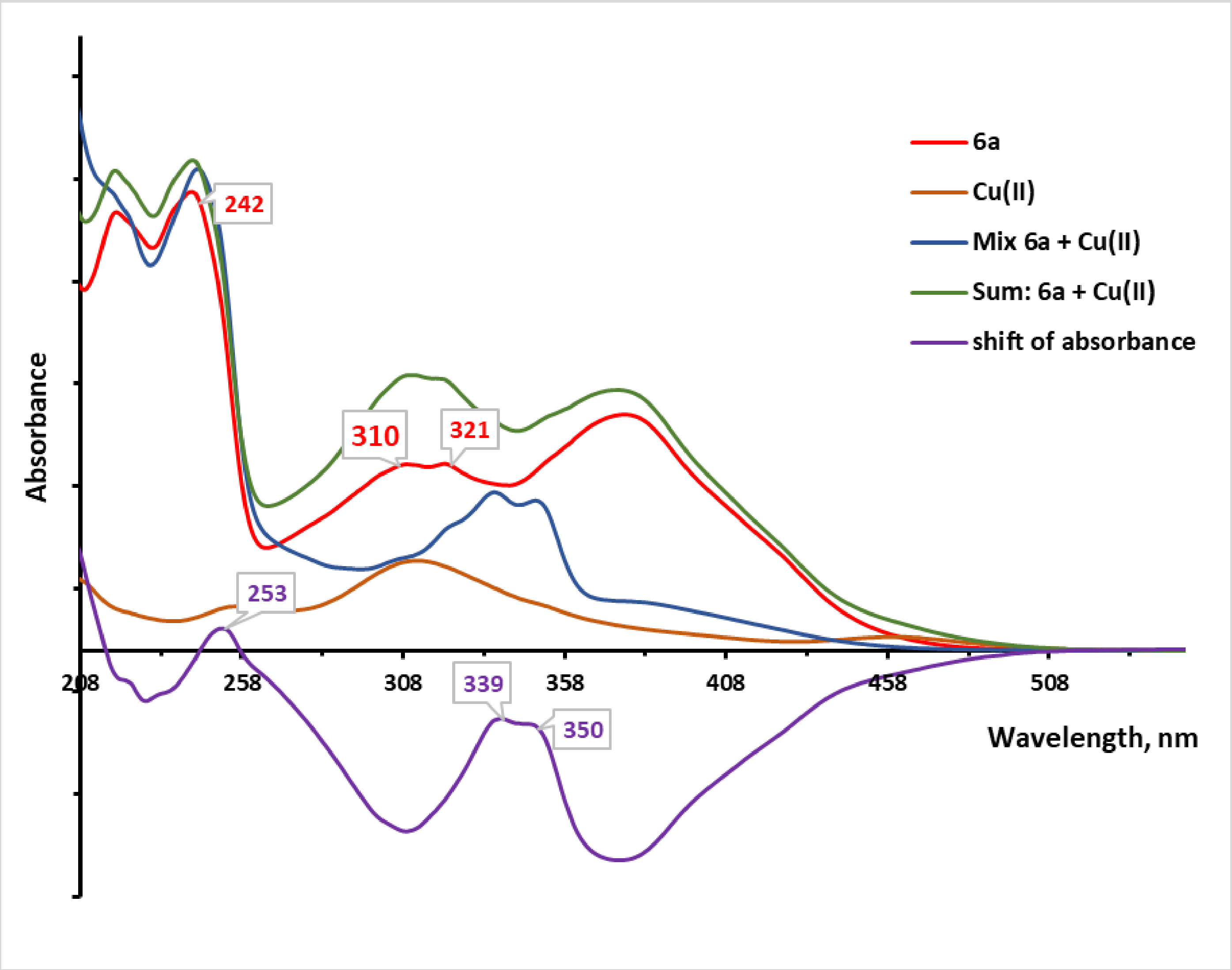

Figure S1. Absorption spectra of compound **6a** (40μM), Cu^{2+} ions solution (40μM), a sum of **6a** and Cu^{2+}, their mixture, and the shift of the spectra caused by the formation of a complex.

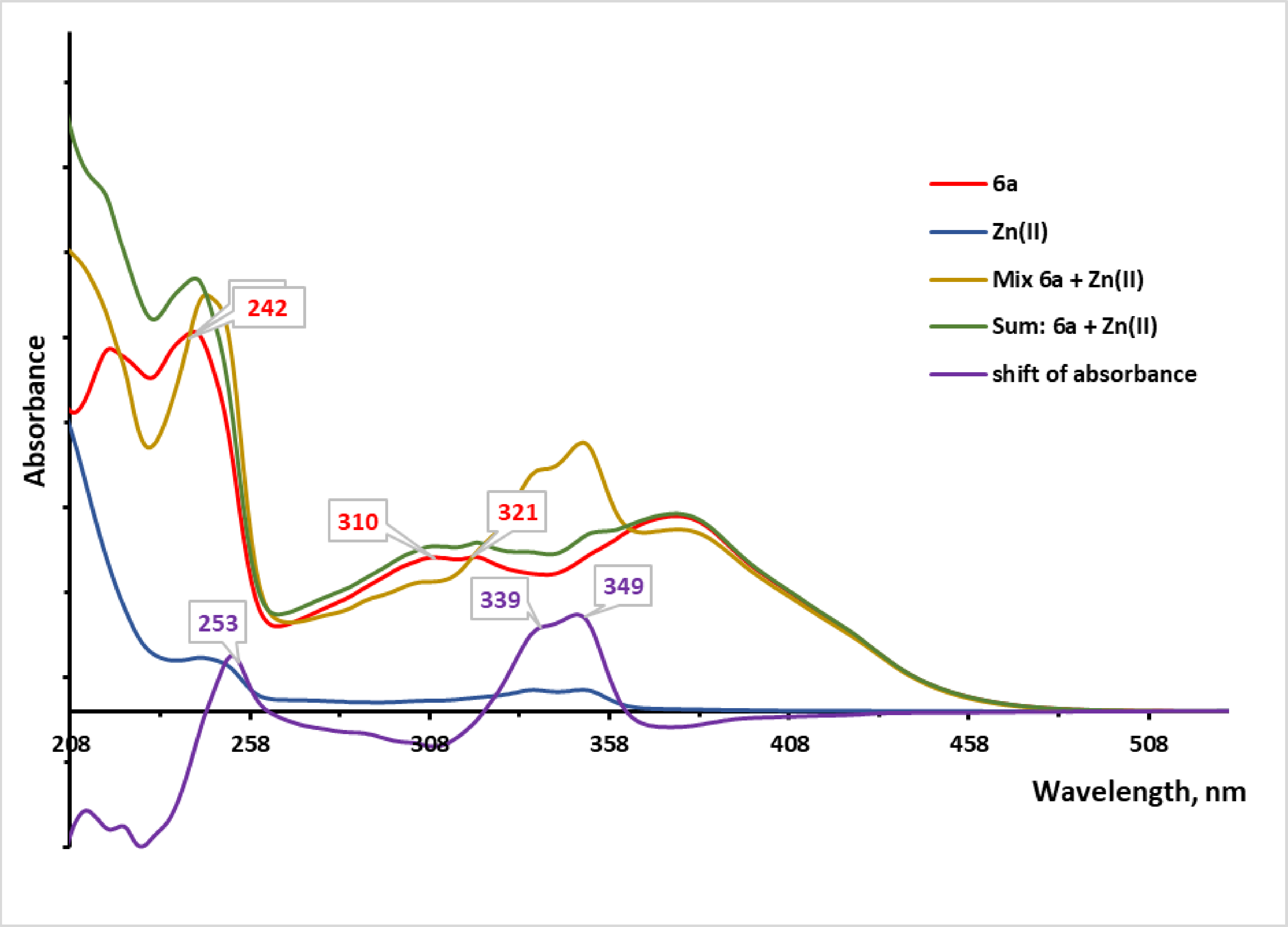

Figure S2. Absorption spectra of compound **6a** (40μM), Zn^{2+} ions solution (40μM), a sum of **6a** and Zn^{2+}, their mixture, and the shift of the spectra caused by the formation of a complex.

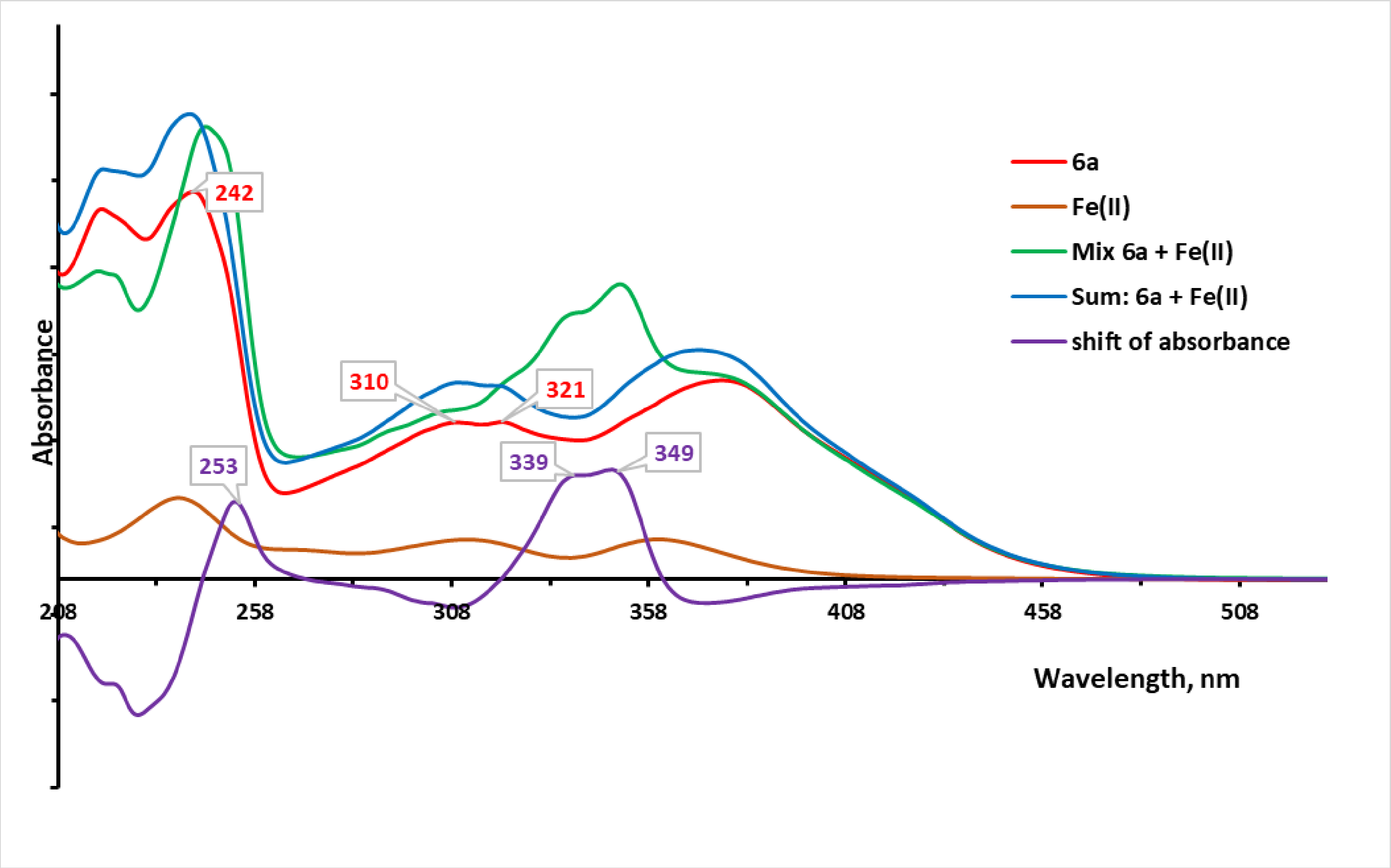

Figure S3. Absorption spectra of compound **6a** (40µM), Fe^{2+} ions solution (40µM), a sum of **6a** and Fe^{2+}, their mixture, and the shift of the spectra caused by the formation of a complex.

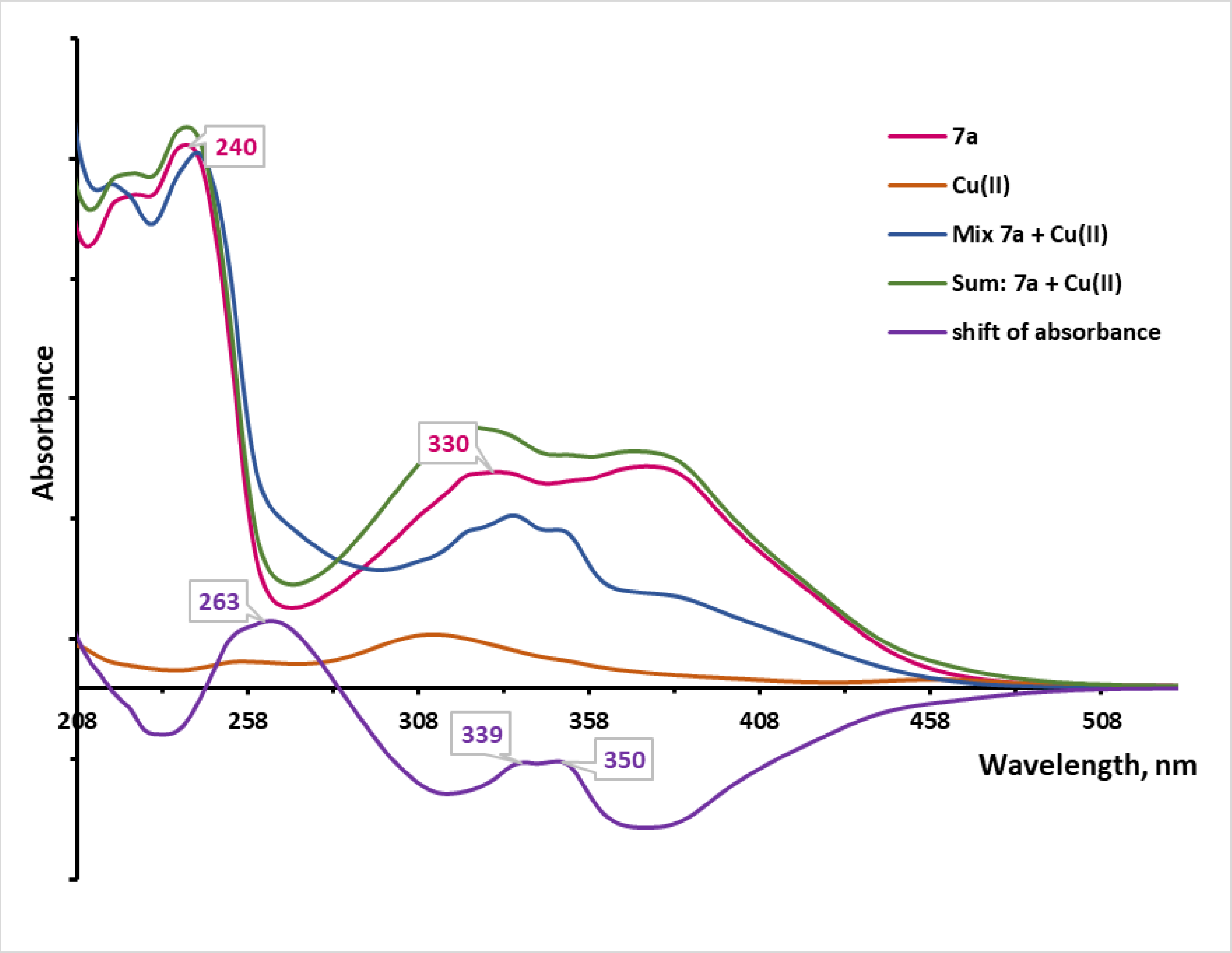

Figure S4. Absorption spectra of compound **7a** (40μM), Cu^{2+} ions solution (40μM), a sum of **7a** and Cu^{2+}, their mixture, and the shift of the spectra caused by the formation of a complex.

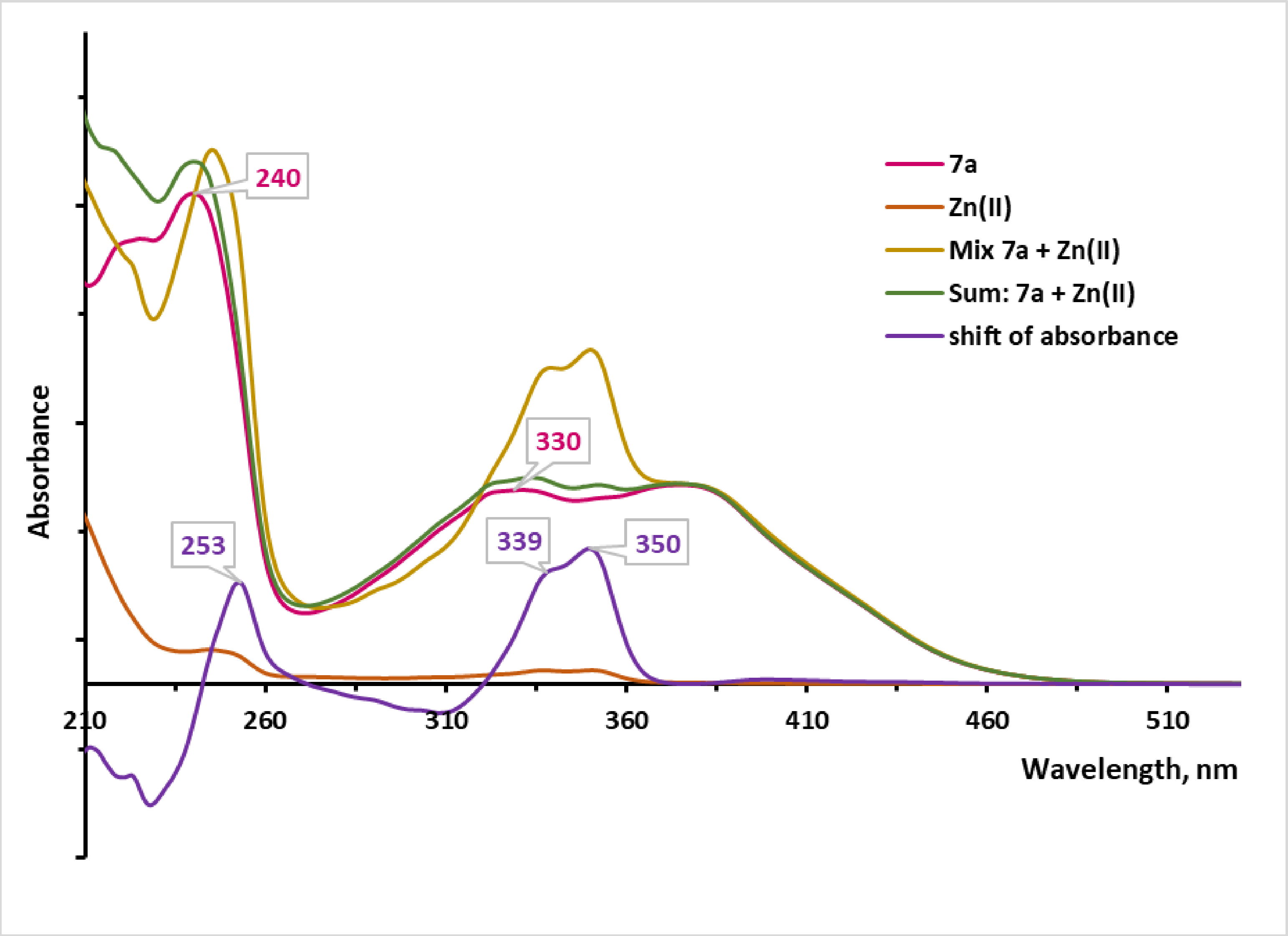

Figure S5. Absorption spectra of compound **7a** (40µM), Zn^{2+} ions solution (40µM), a sum of **7a** and Zn^{2+}, their mixture, and the shift of the spectra caused by the formation of a complex.

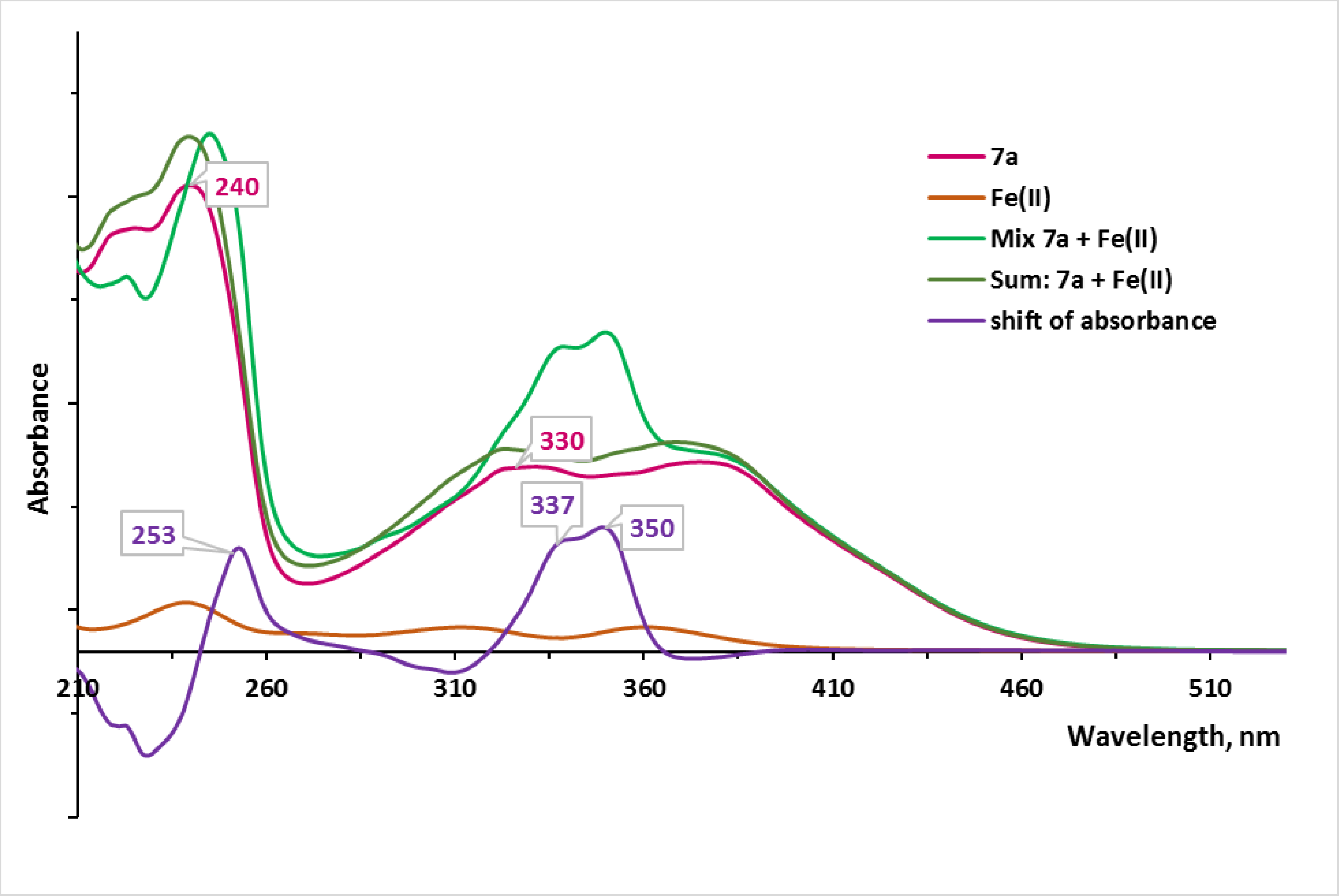

Figure S6. Absorption spectra of compound **7a** (40μM), Fe^{2+} ions solution (40μM), a sum of **7a** and Fe^{2+}, their mixture, and the shift of the spectra caused by the formation of a complex.

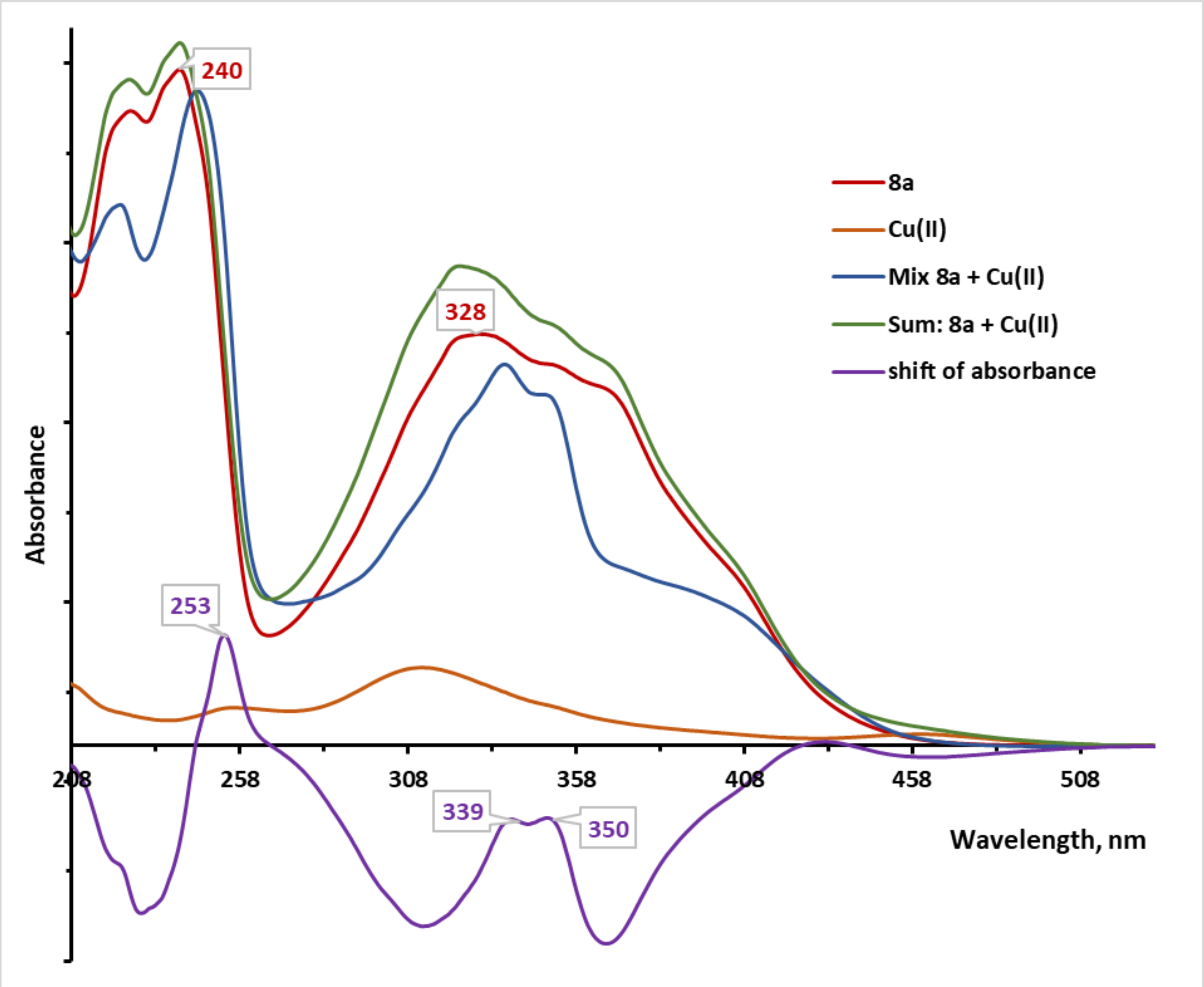

Figure S7. Absorption spectra of compound **8a** (40μM), Cu^{2+} ions solution (40μM), a sum of **8a** and Cu^{2+}, their mixture, and the shift of the spectra caused by the formation of a complex

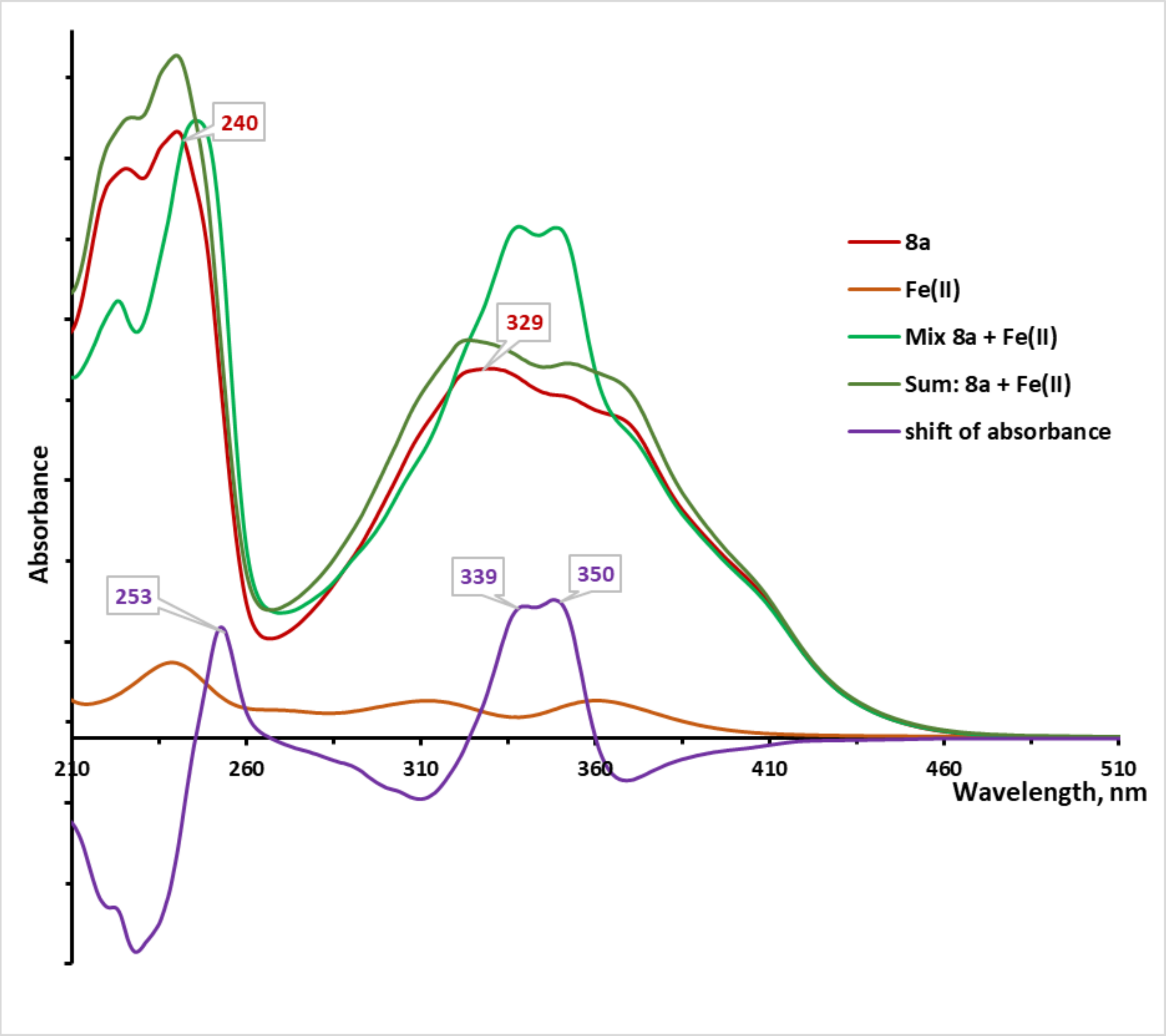

Figure S8. Absorption spectra of compound **8a** (40μM), Fe^{2+} ions solution (40μM), a sum of **8a** and Fe^{2+}, their mixture, and the shift of the spectra caused by the formation of a complex

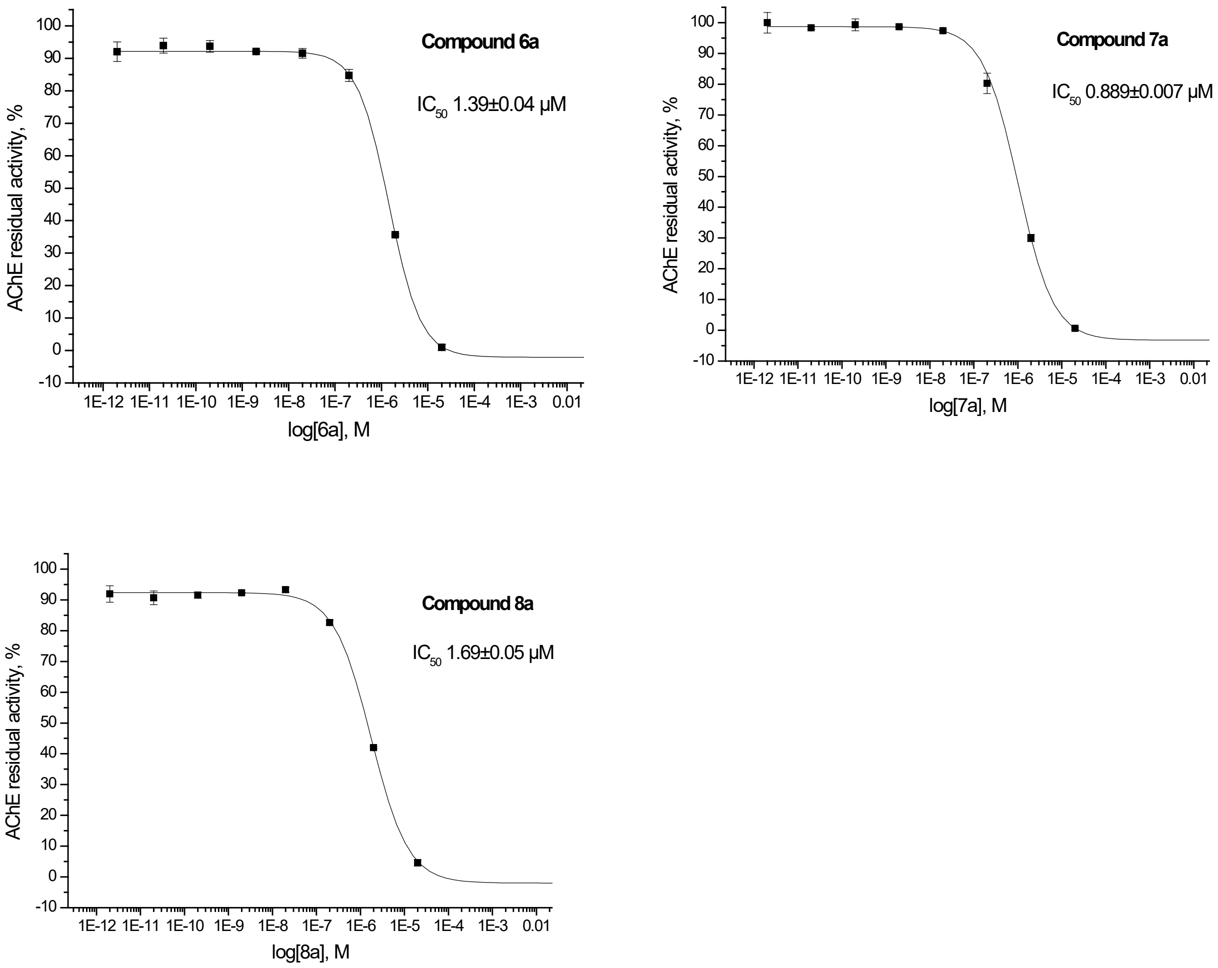

Figure S9. IC_{50} values for AChE inhibition by compounds **6a,7a,8a** (MEAN ± SEM, n = 3)

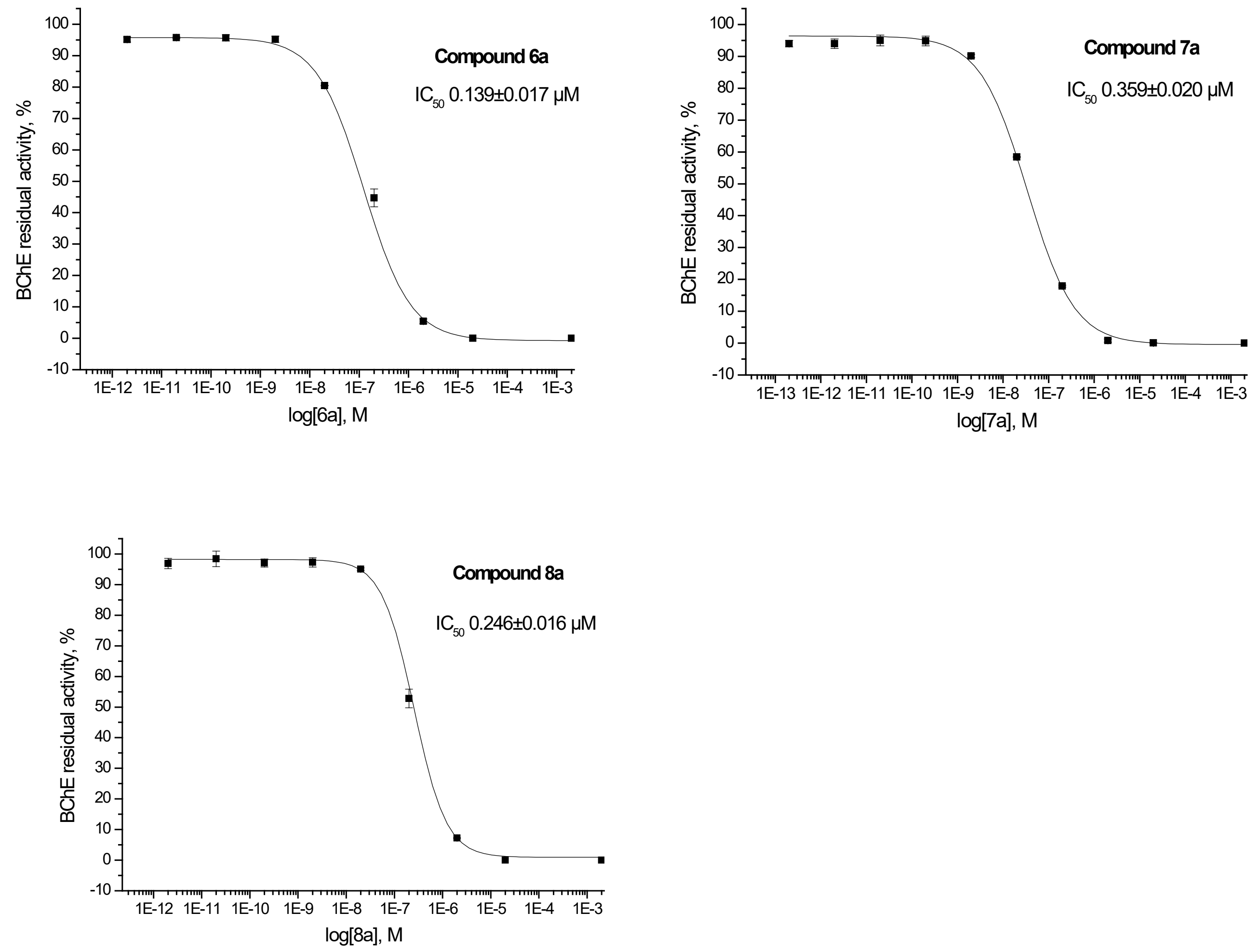

Figure S10. IC_{50} values for BChE inhibition by compounds **6a,7a,8a** (MEAN ± SEM, n = 3)

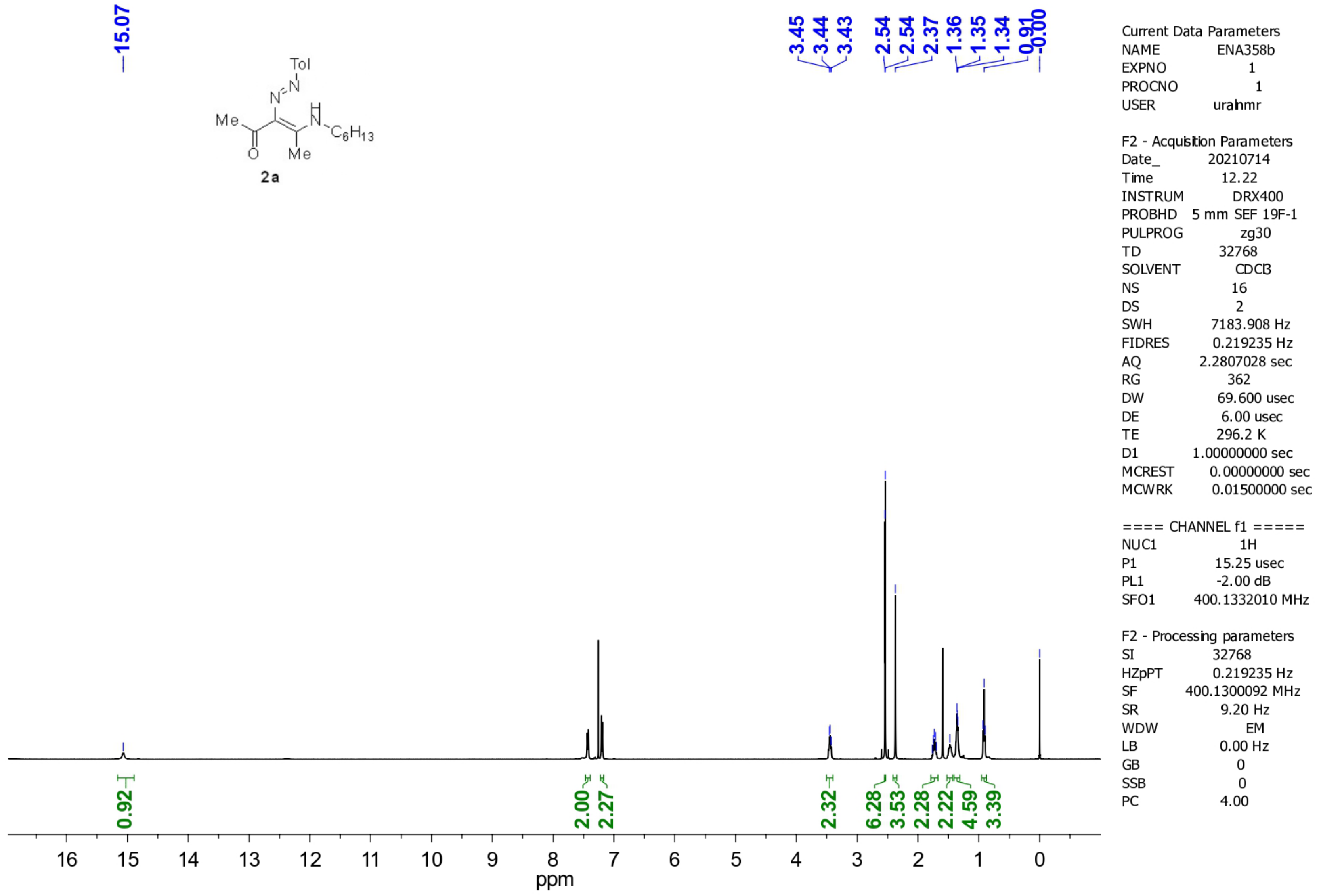

Figure S11. ^{1}H NMR spectrum of compound **2a**

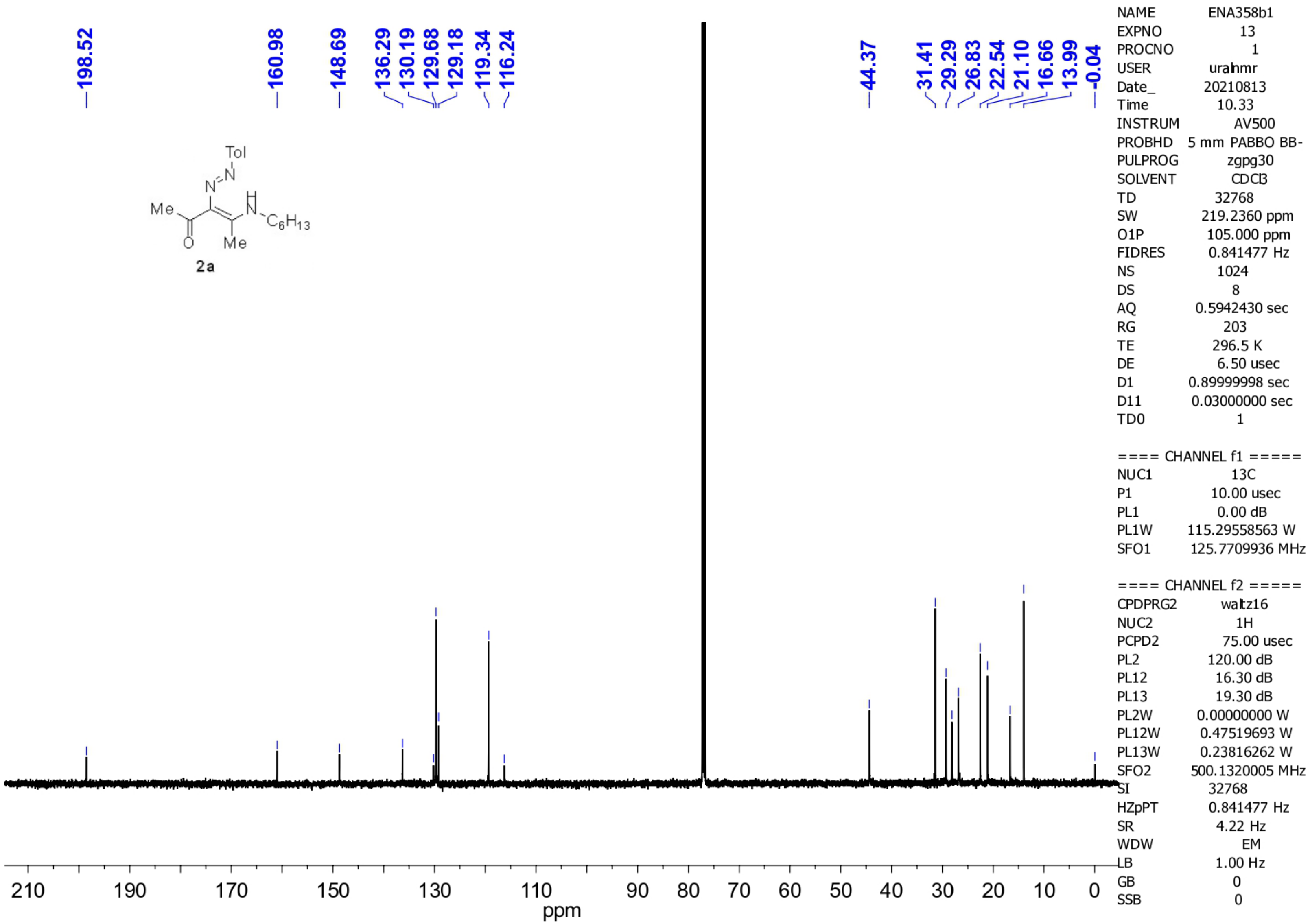

Figure S12. ^{13}C NMR spectrum of compound **2a**

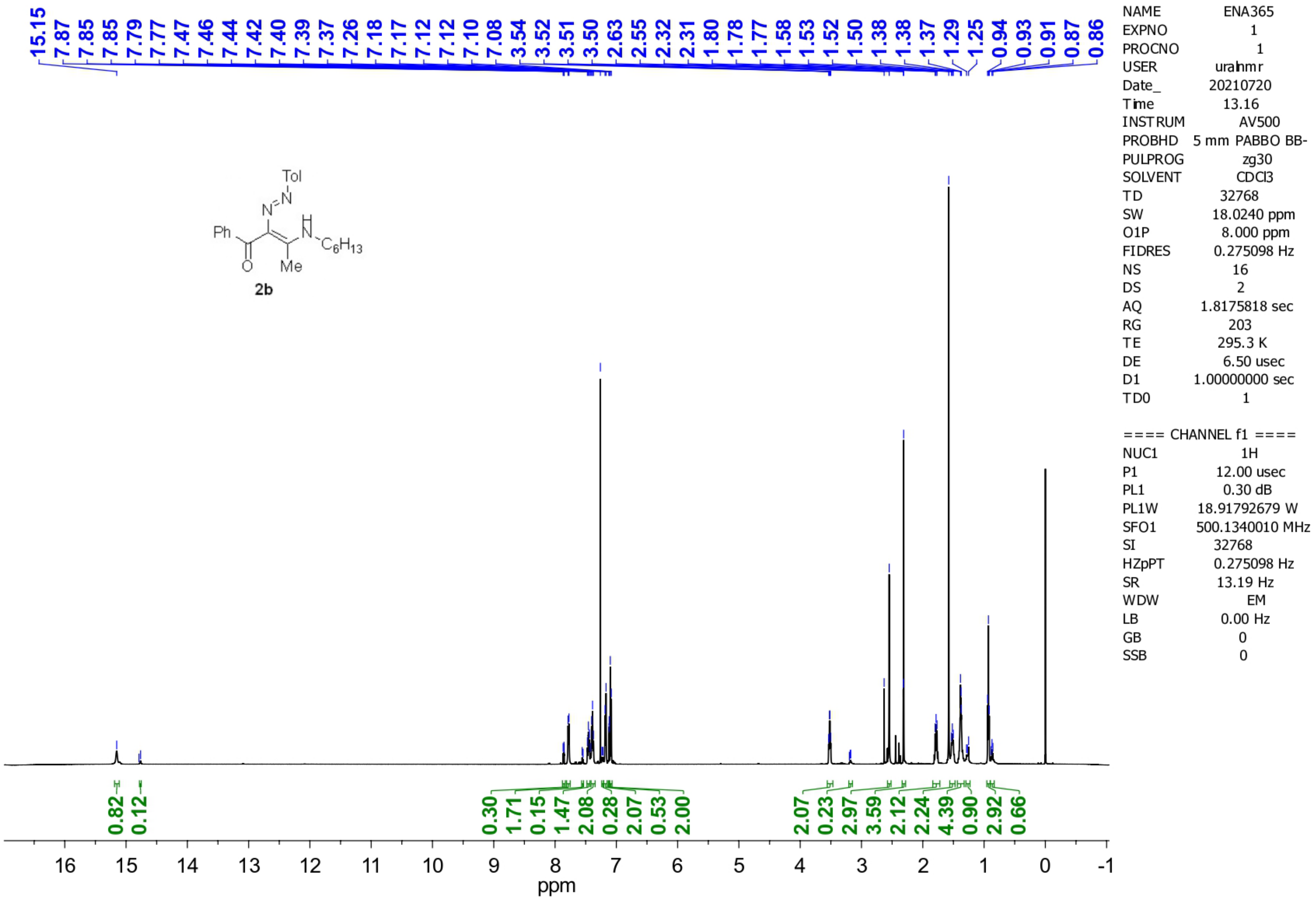

Figure S13. ^{1}H NMR spectrum of compound **2b**

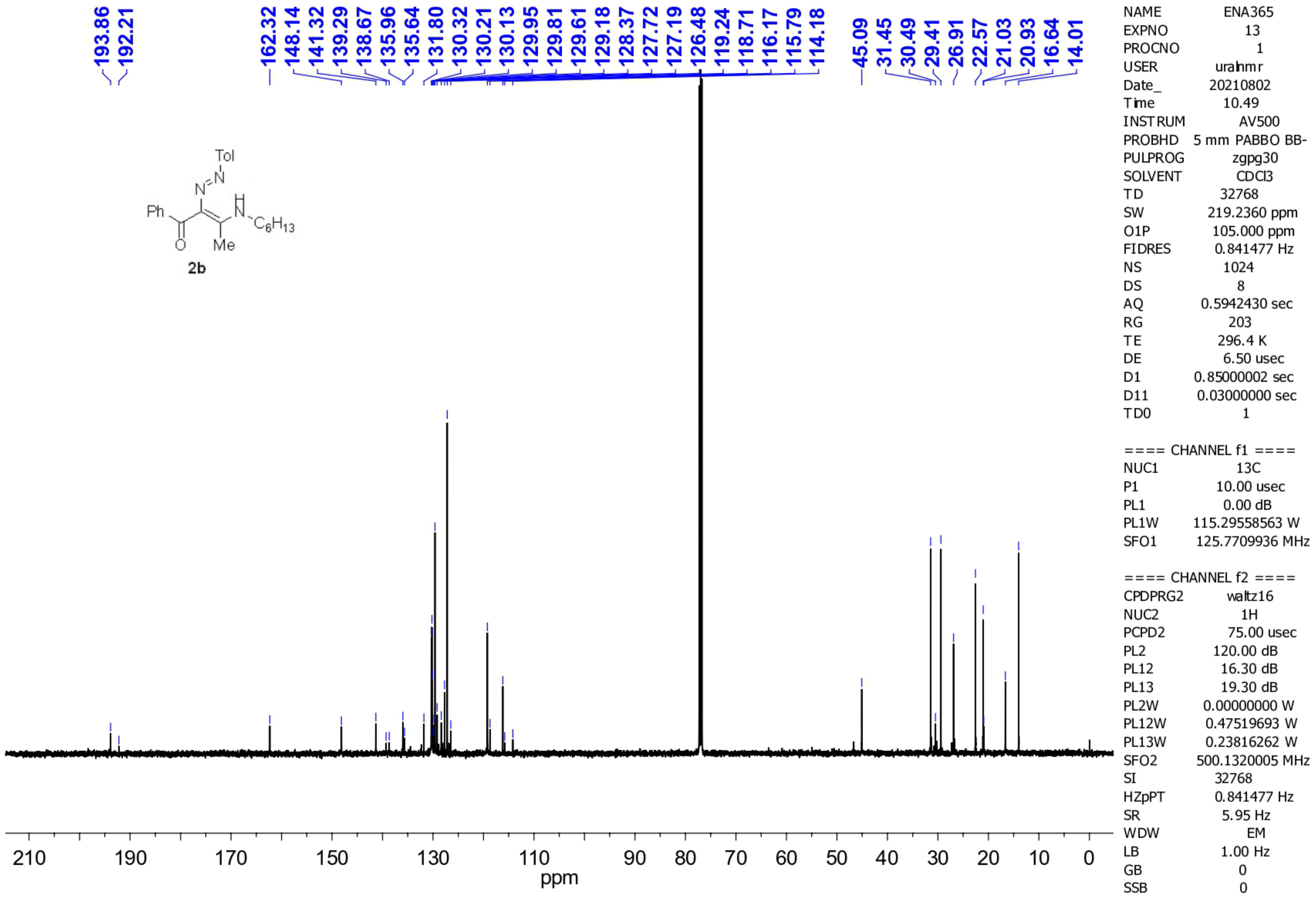

Figure S14. ^{13}C NMR spectrum of compound **2b**

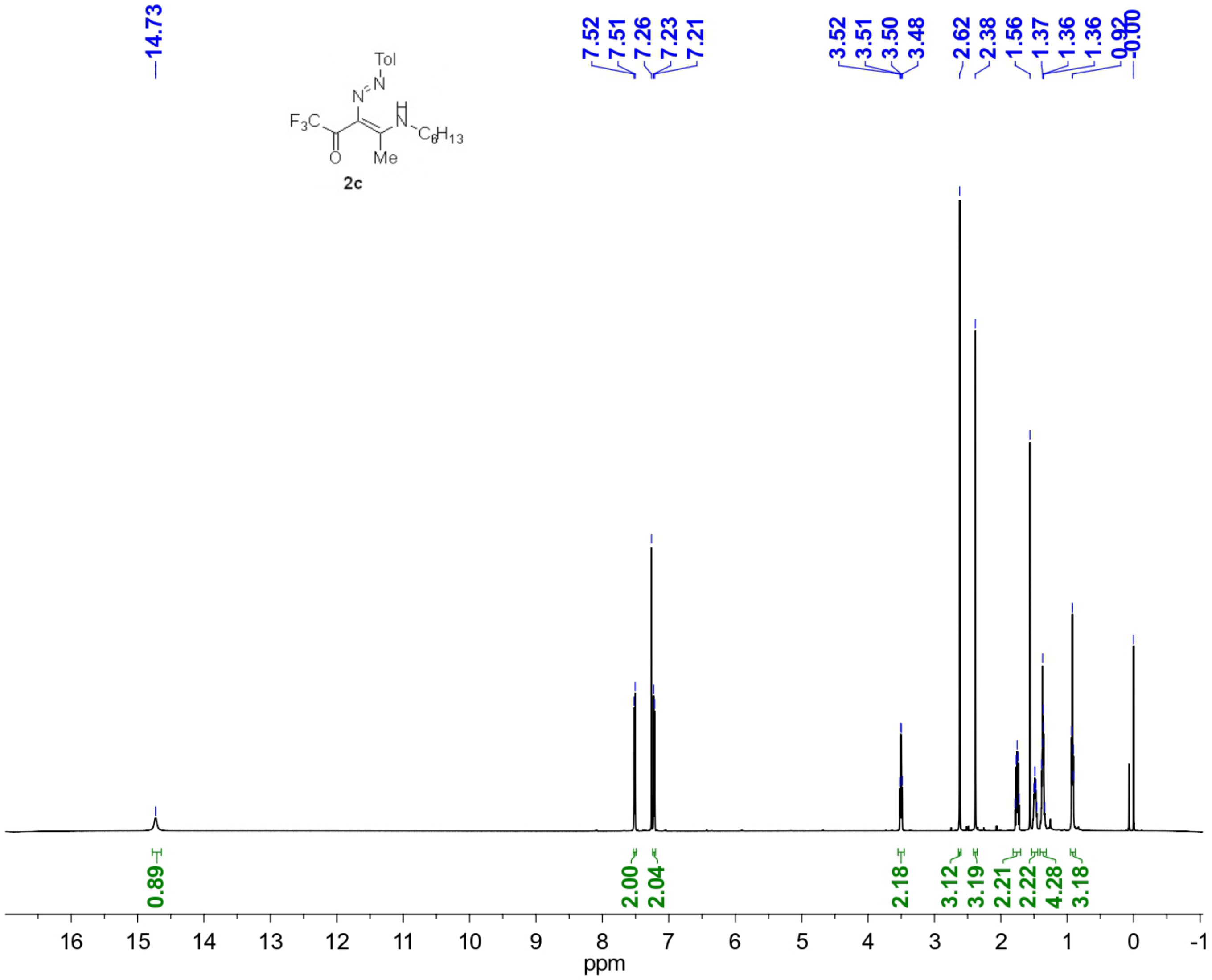

Figure S15. ¹H NMR spectrum of compound **2c**

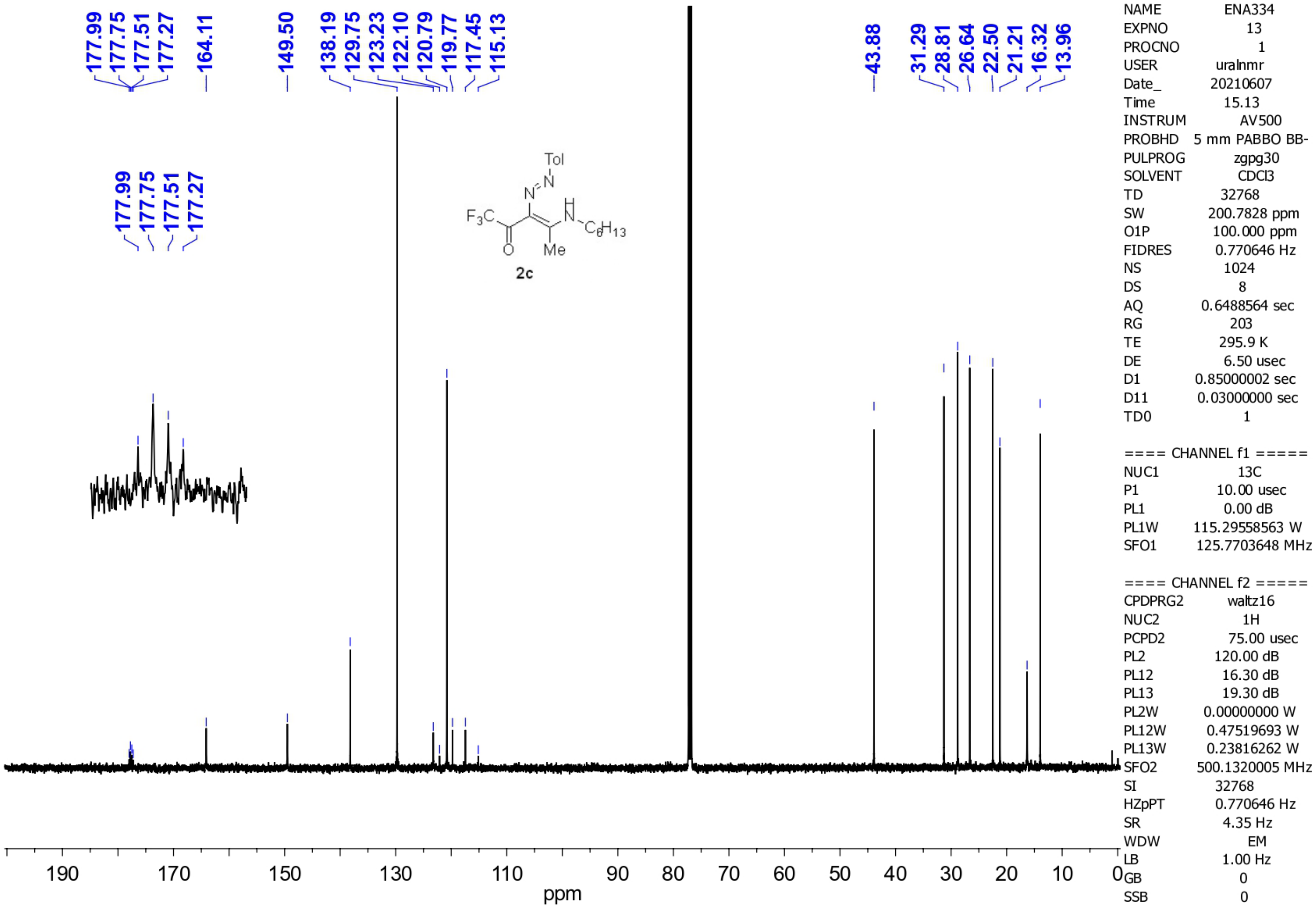

Figure S16. ^{13}C NMR spectrum of compound **2c**

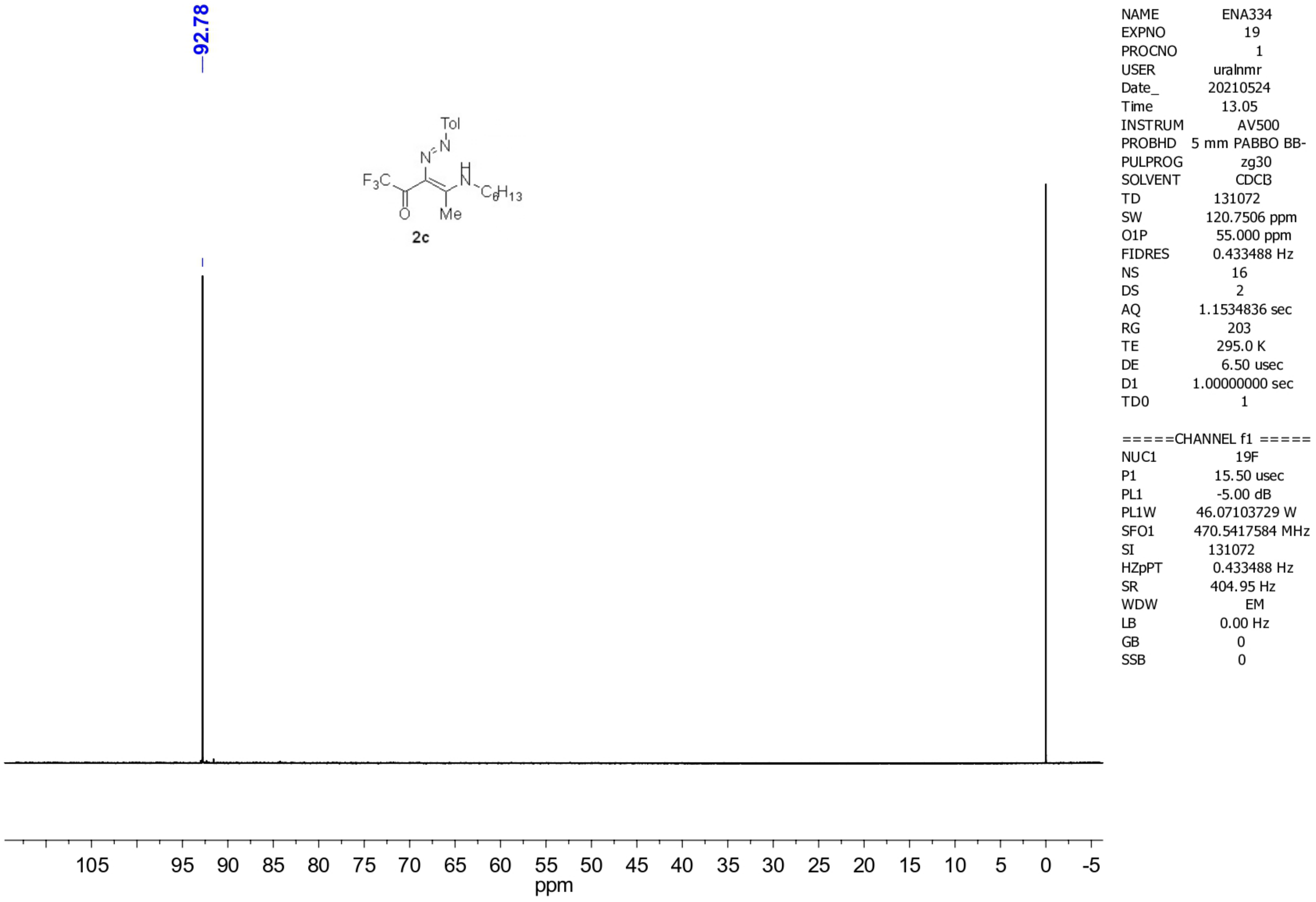

Figure S17. ^{19}F NMR spectrum of compound **2c**

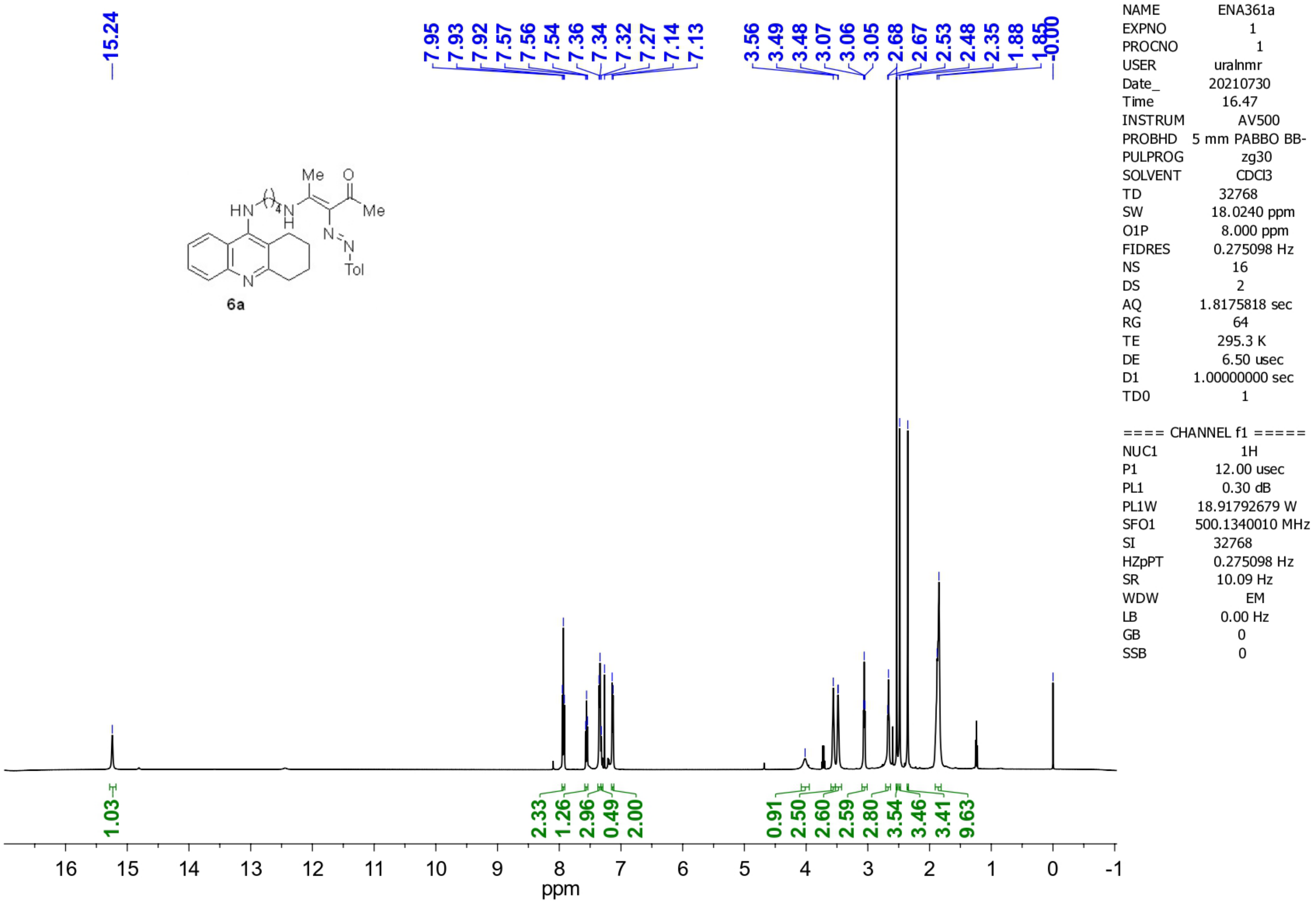

Figure S18 ^{1}H NMR spectrum of compound **6a**

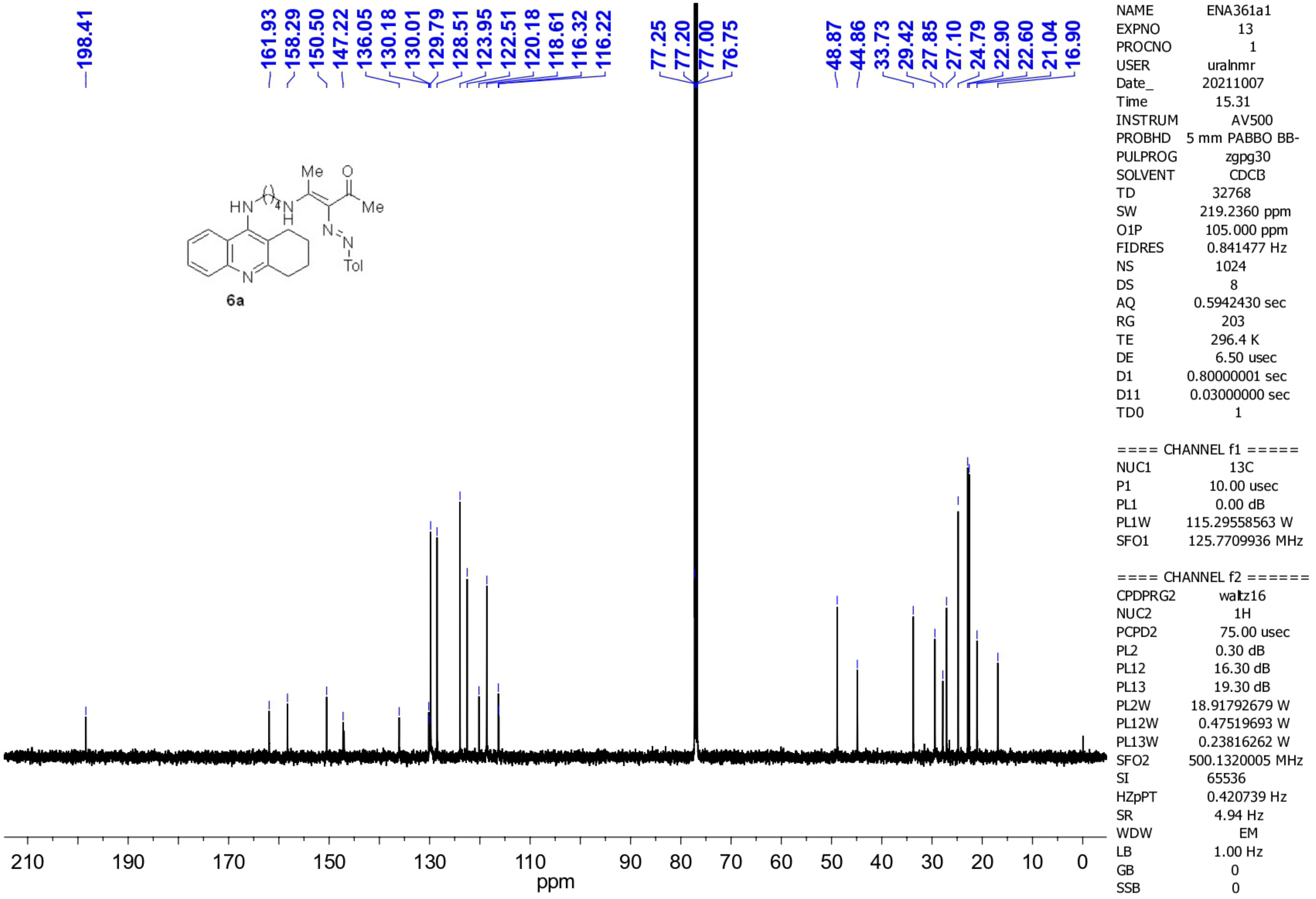

Figure S19. ^{13}C NMR spectrum of compound **6a**

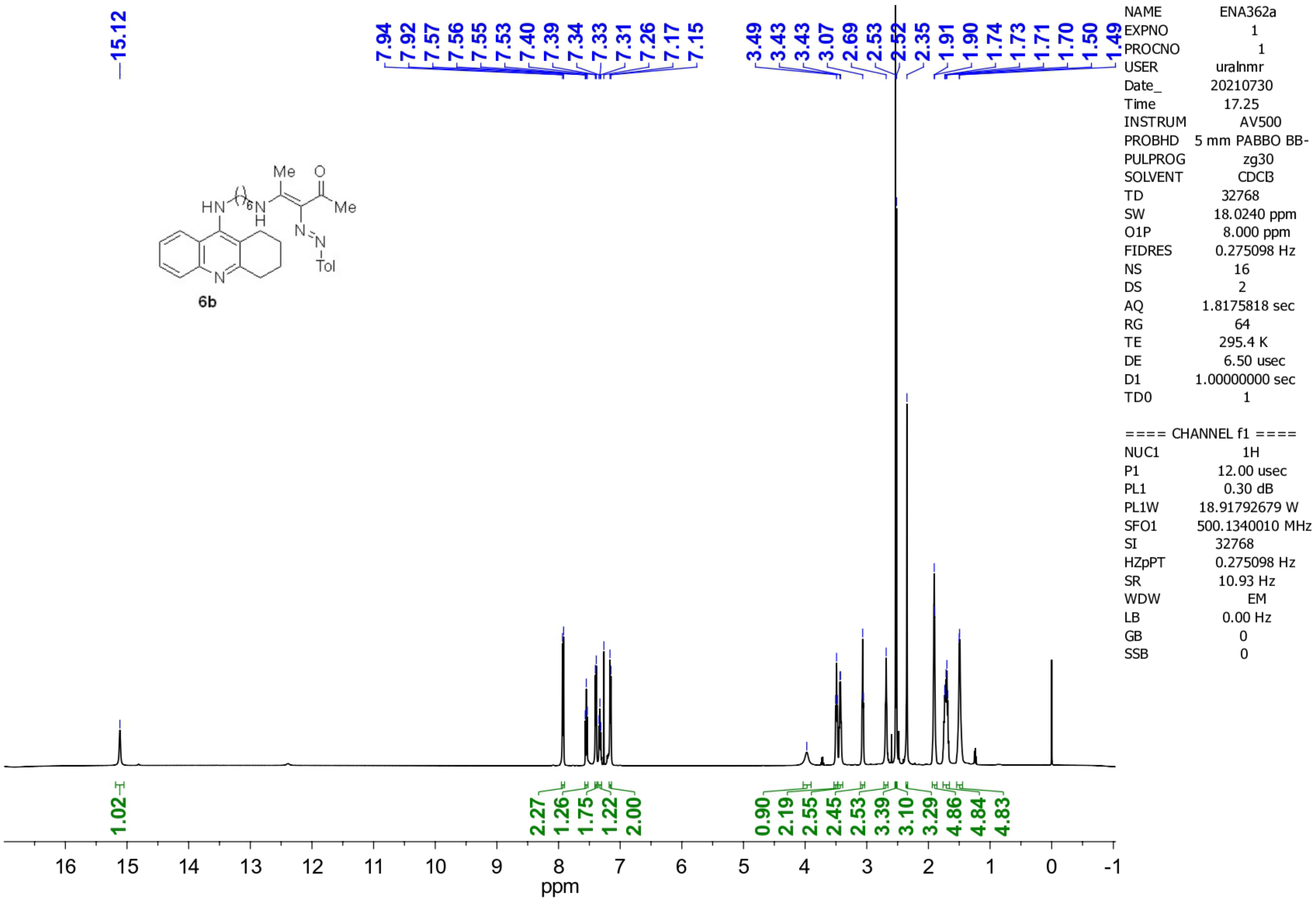

Figure S20. ^{1}H NMR spectrum of compound **6b**

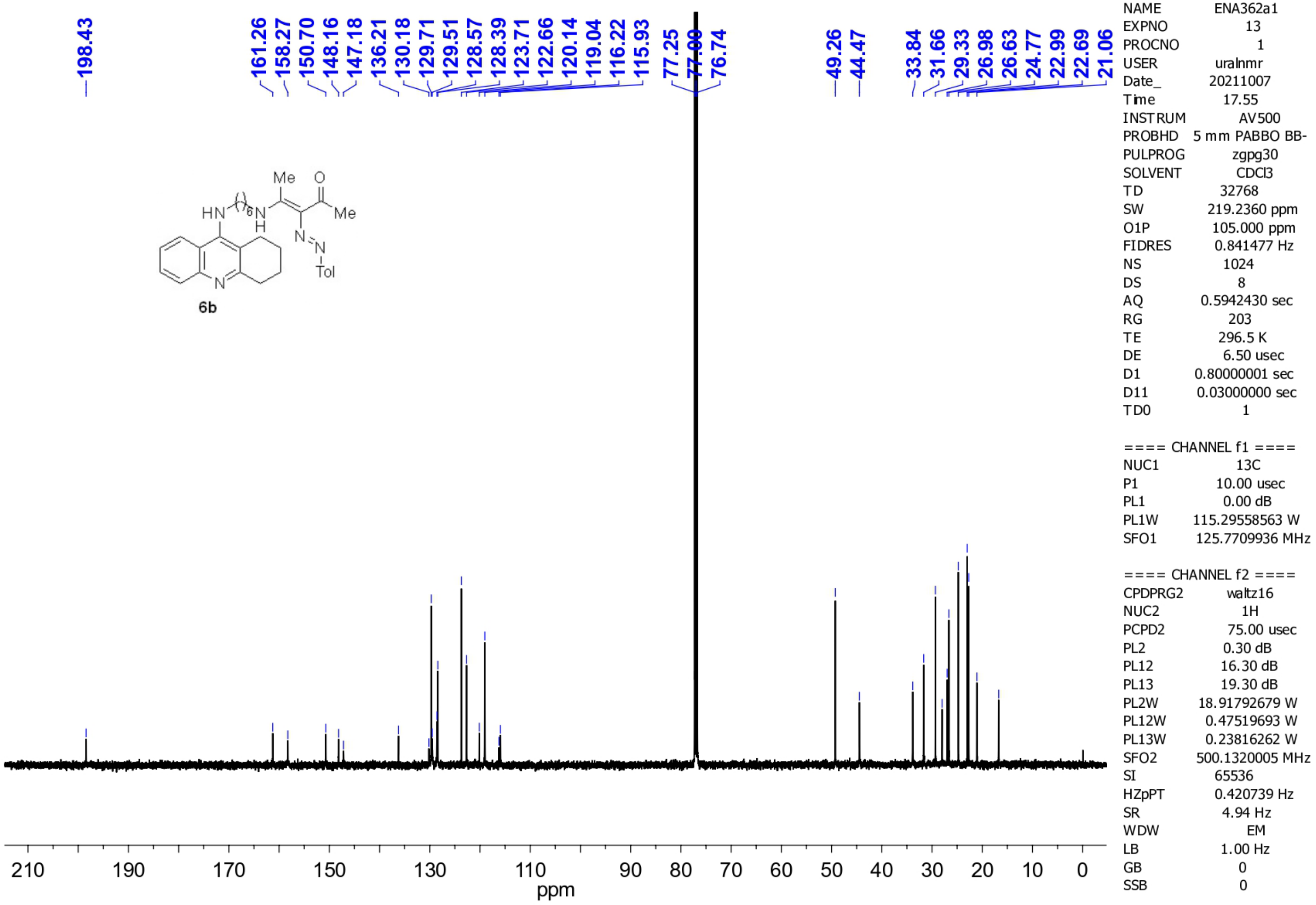

Figure S21. ^{13}C NMR spectrum of compound **6b**

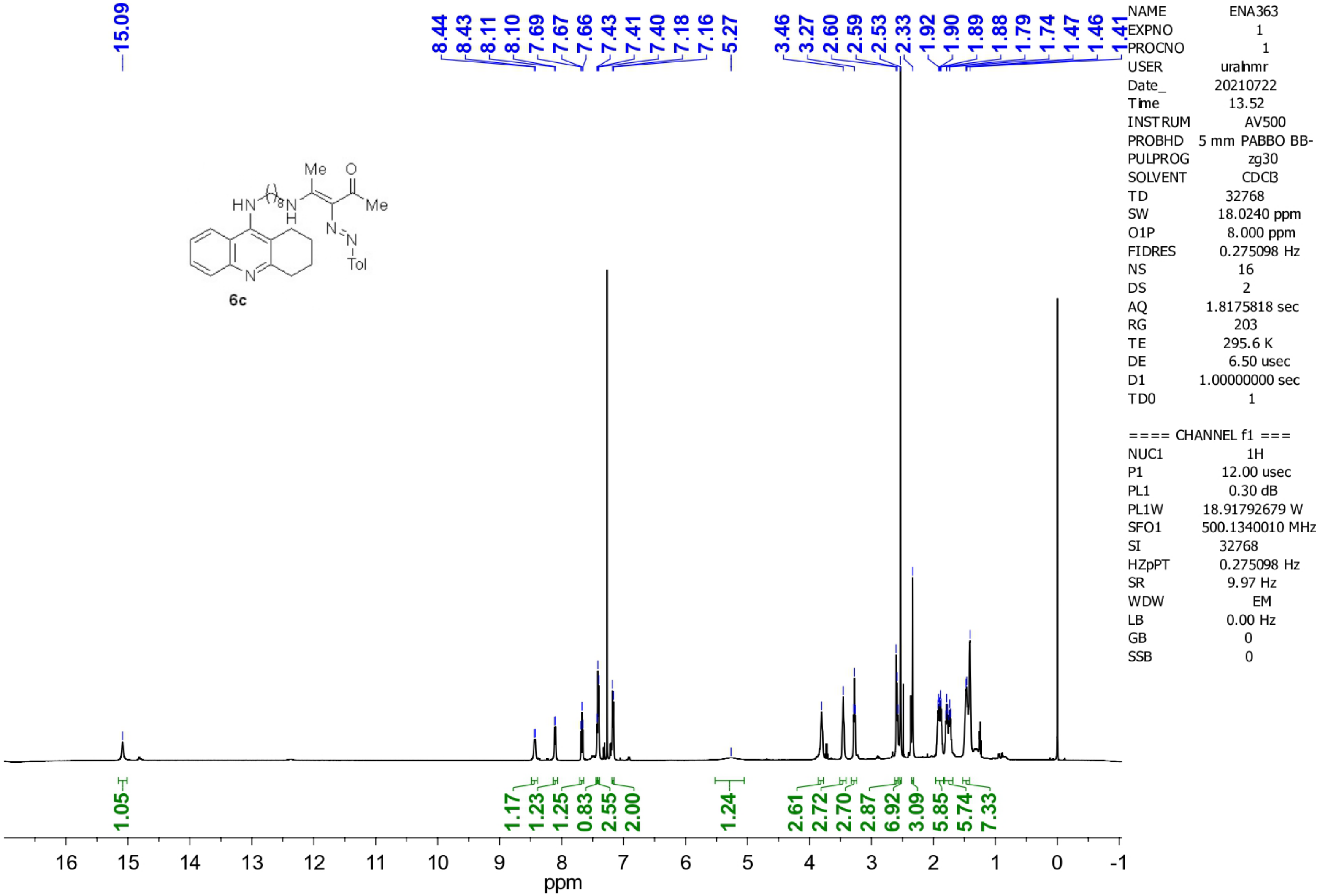

Figure S22. ^{1}H NMR spectrum of compound **6c**

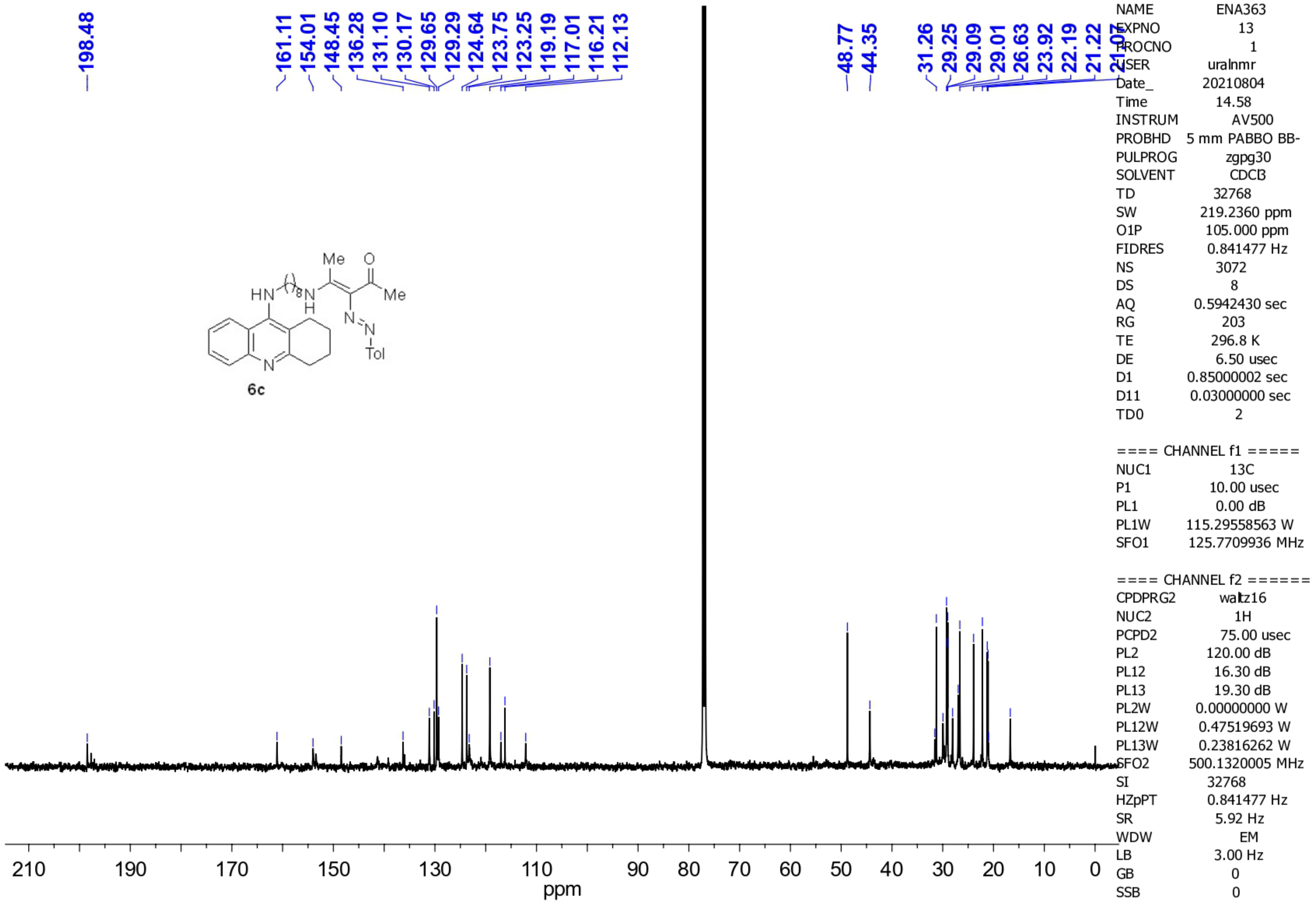

Figure S23. ^{13}C NMR spectrum of compound **6c**

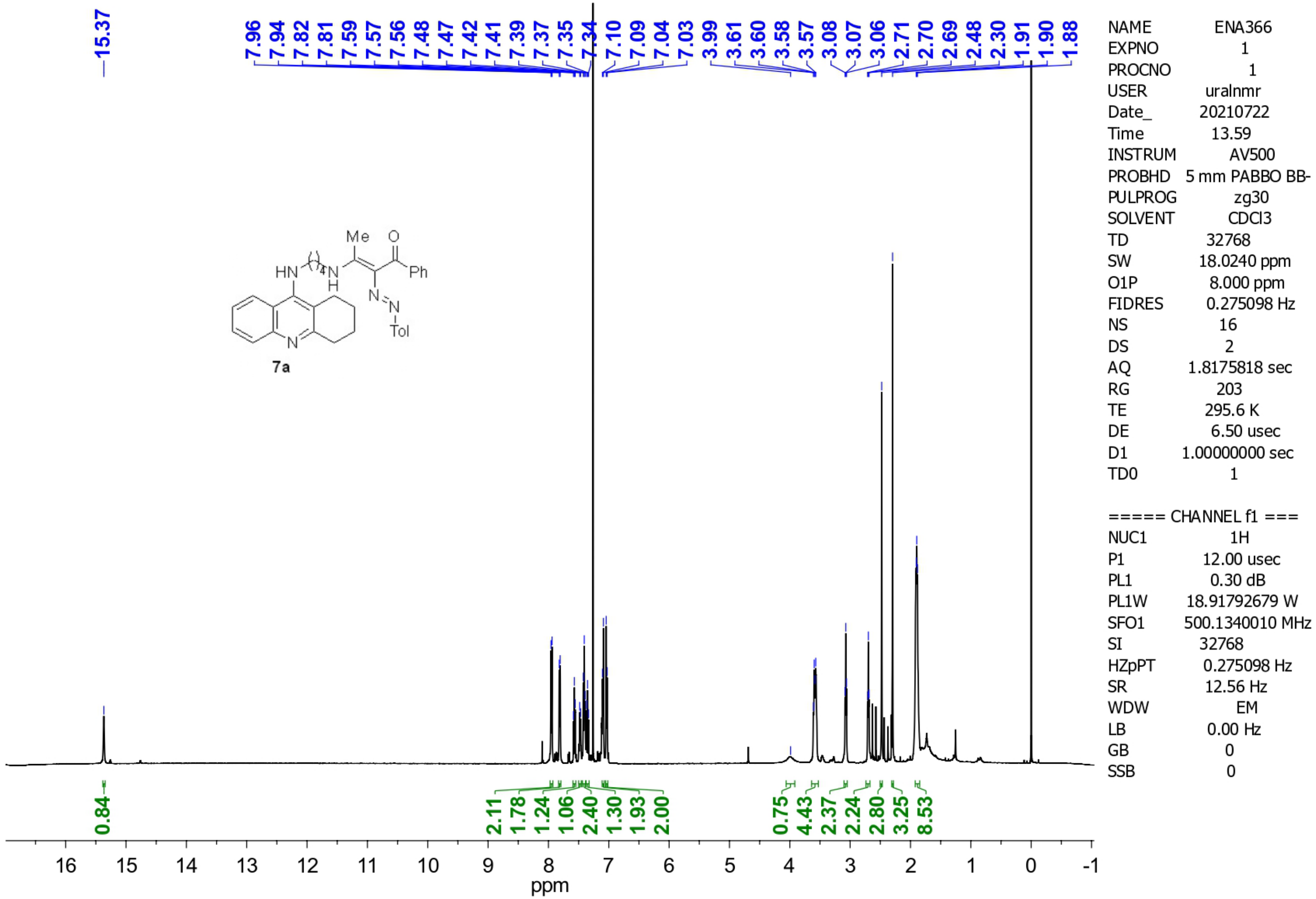

Figure S24. ¹H NMR spectrum of compound **7a**

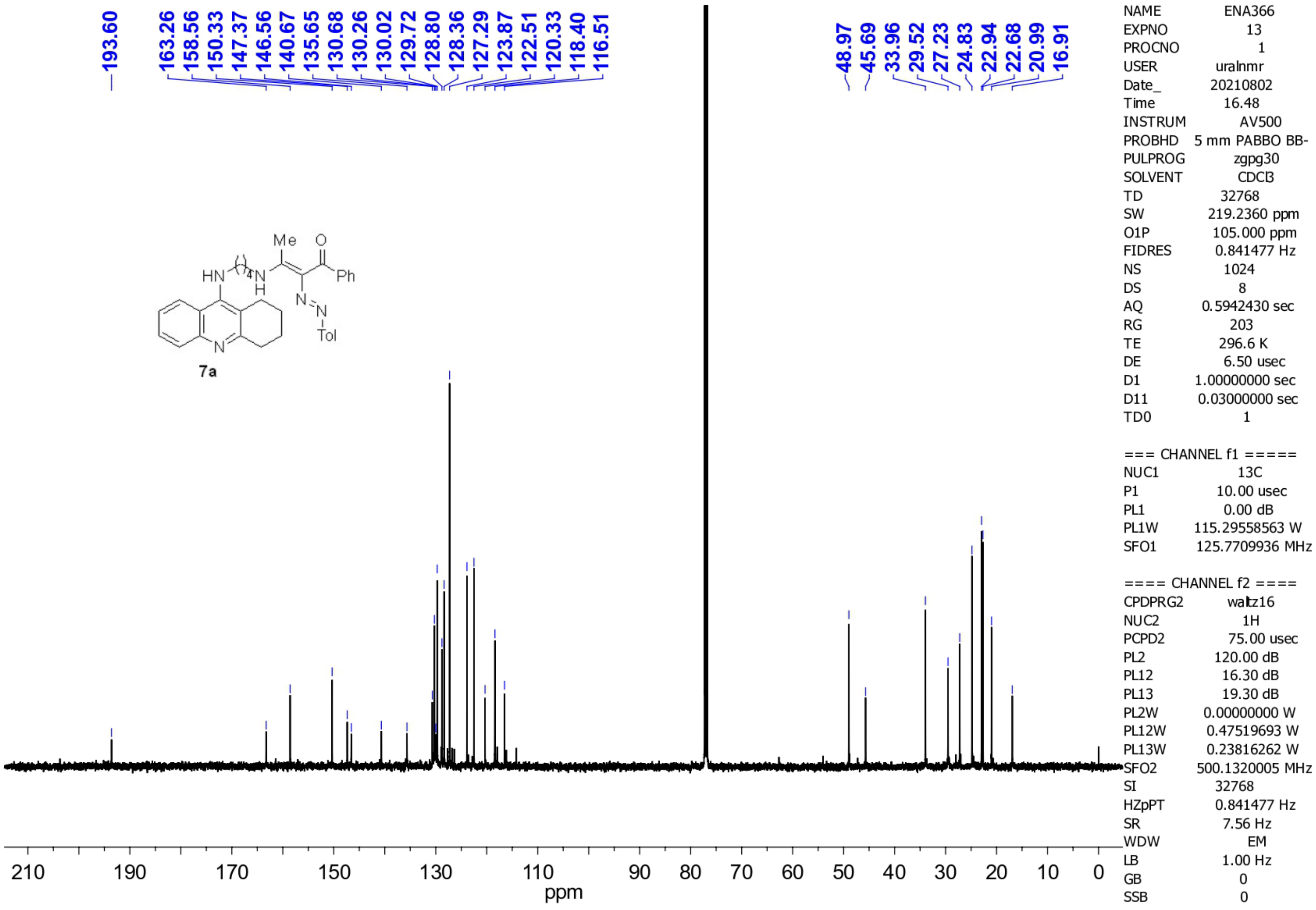

Figure S25. ^{13}C NMR spectrum of compound **7a**

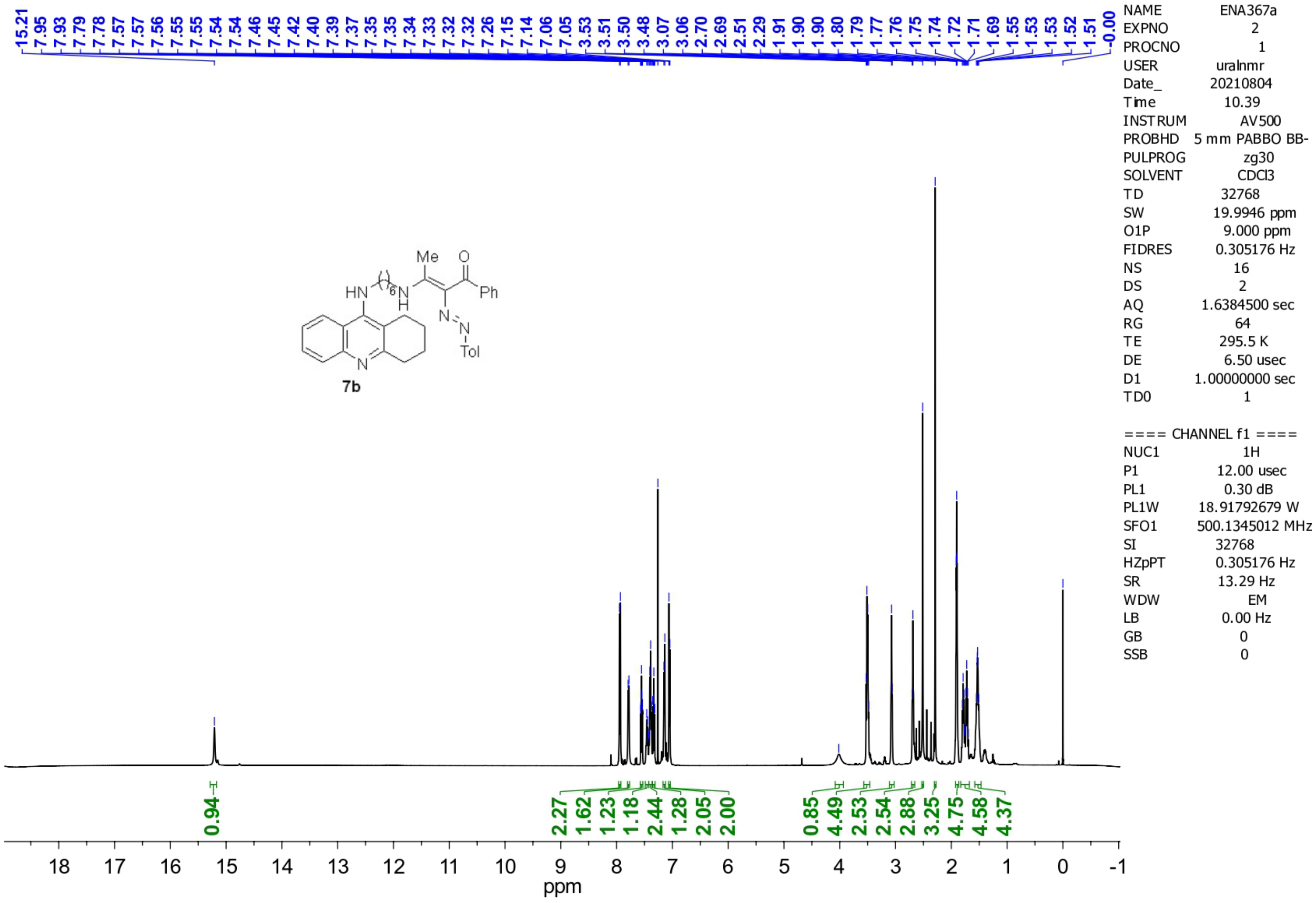

Figure S26. ^{1}H NMR spectrum of compound **7b**

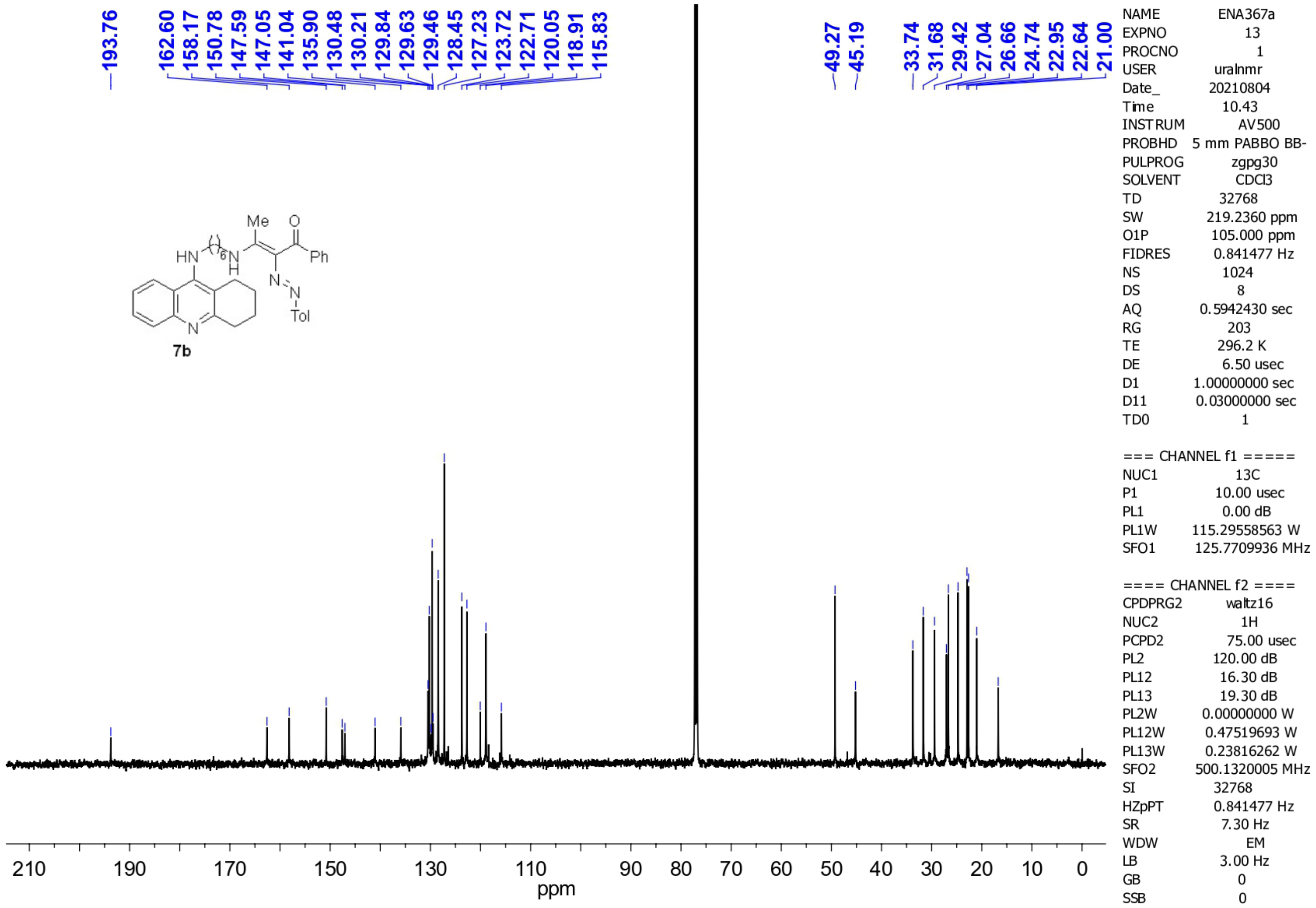

Figure S27. ^{13}C NMR spectrum of compound **7b**

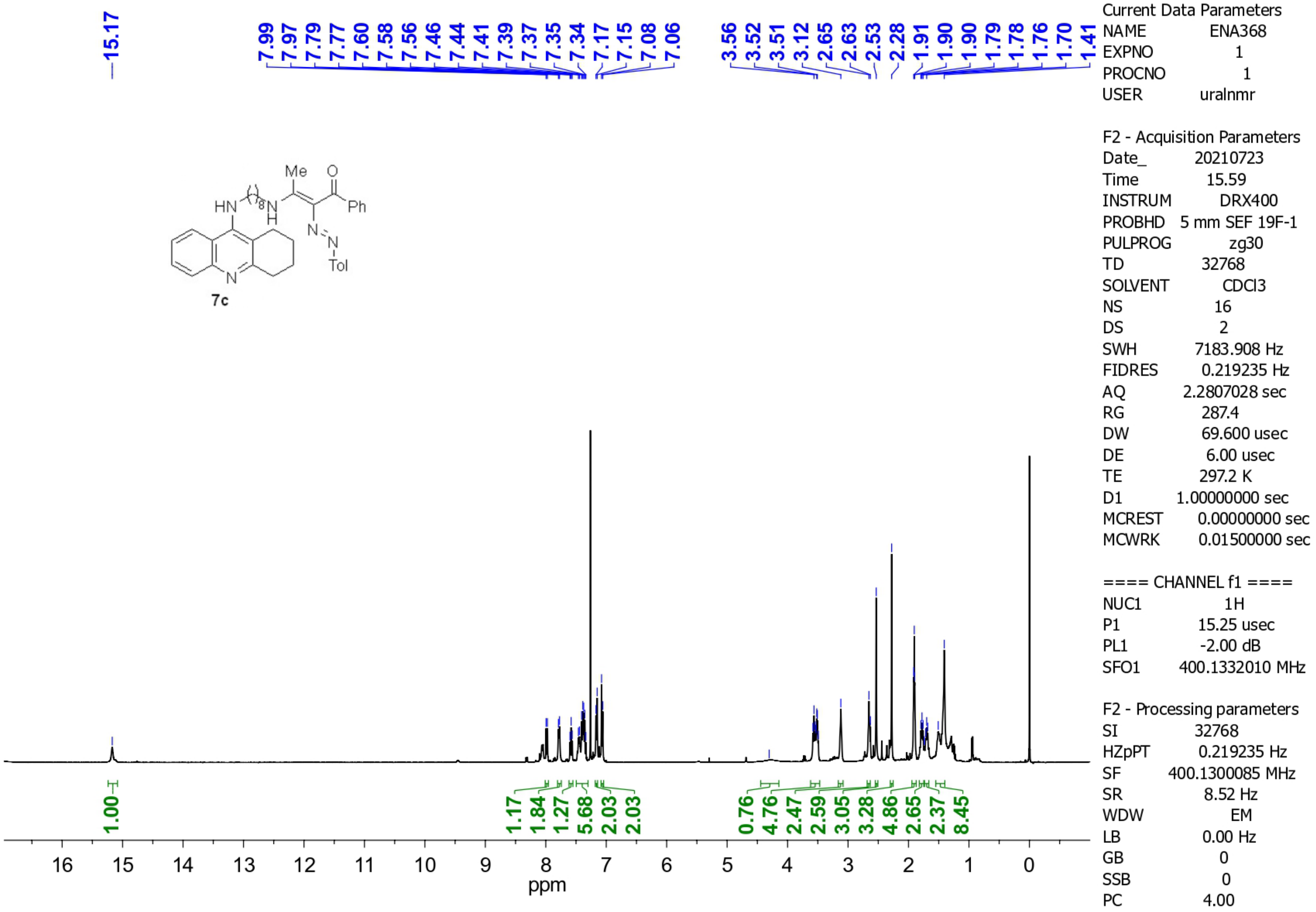

Figure S28. ¹H NMR spectrum of compound **7c**

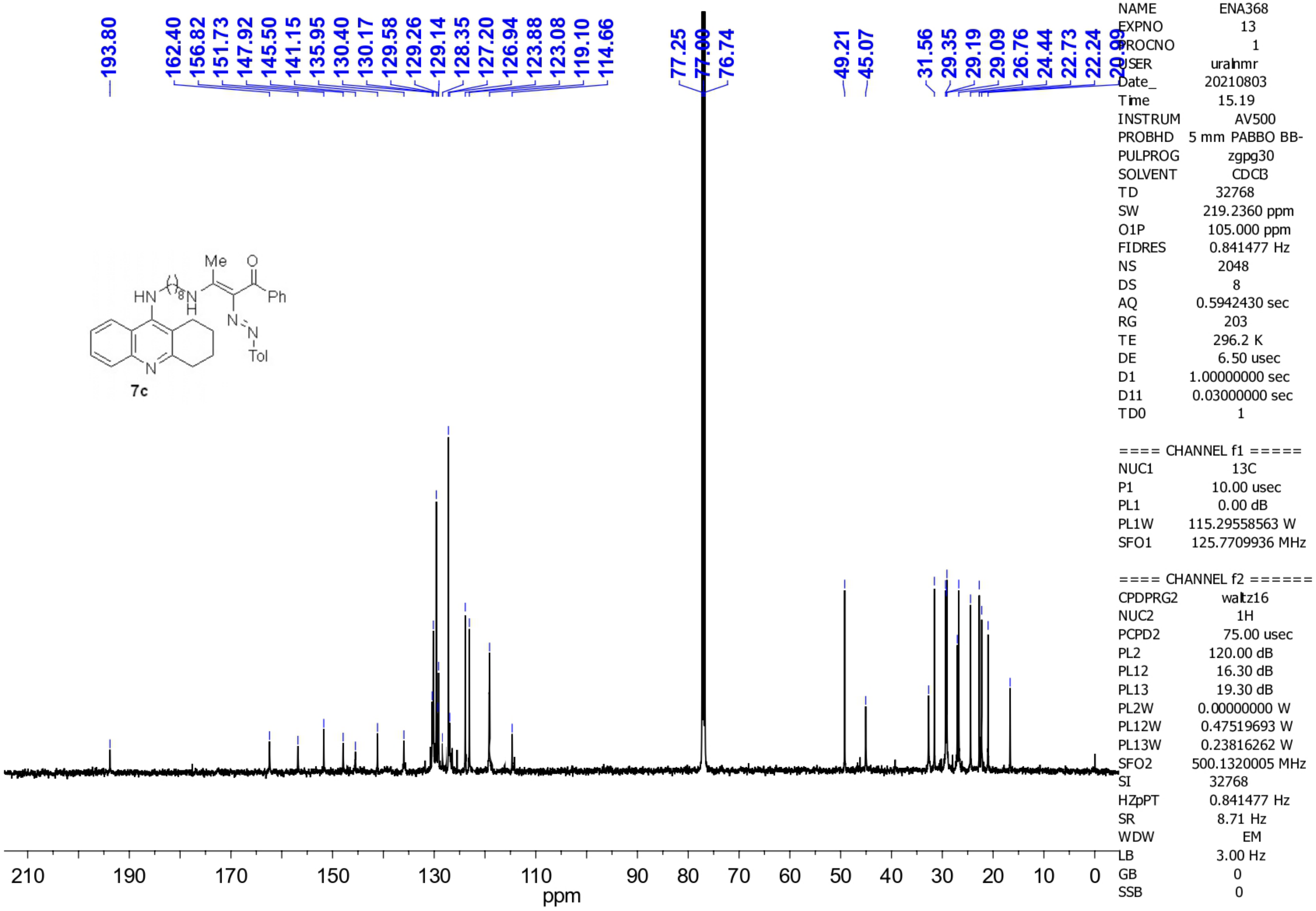

Figure S29. ^{13}C NMR spectrum of compound **7c**

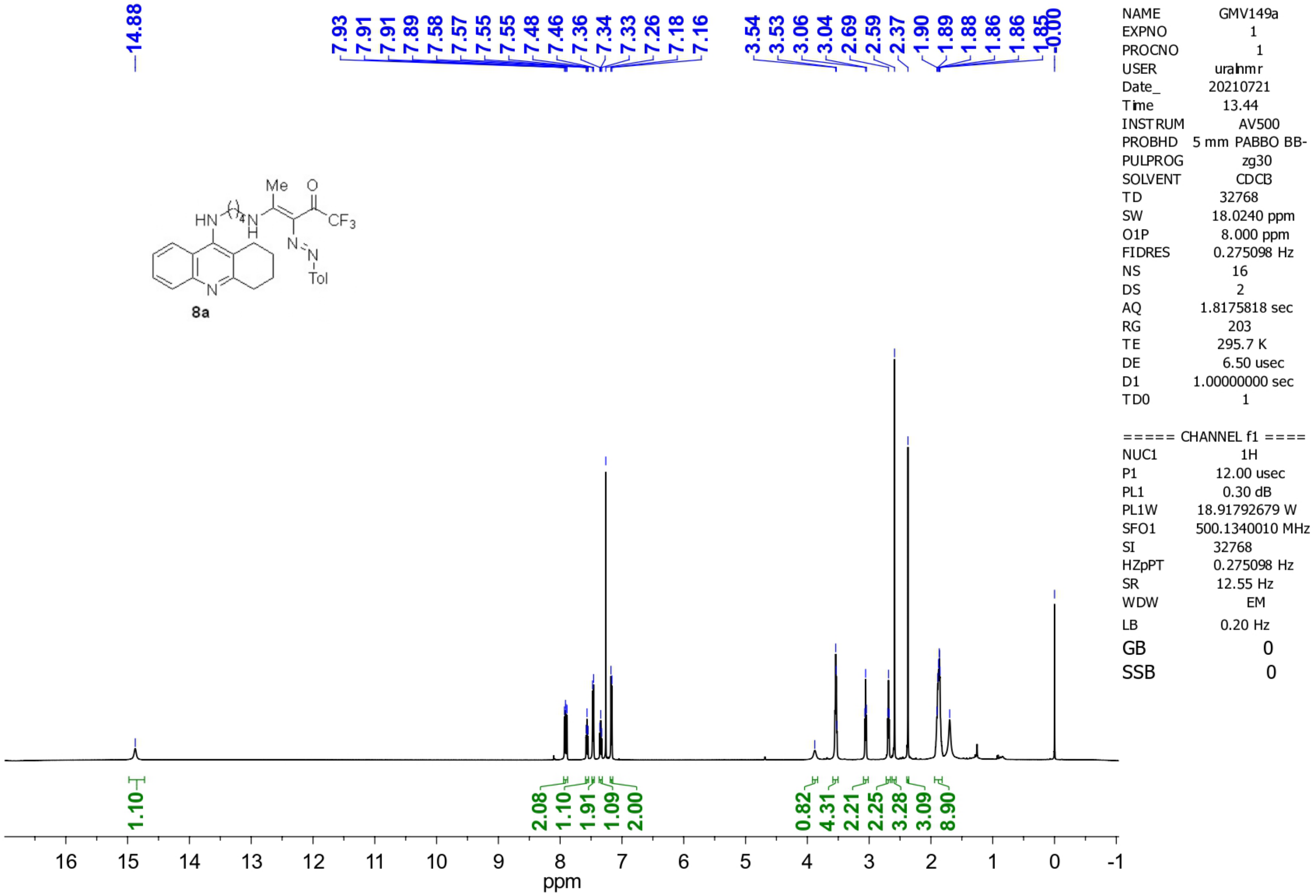

Figure S30. ^{1}H NMR spectrum of compound **8a**

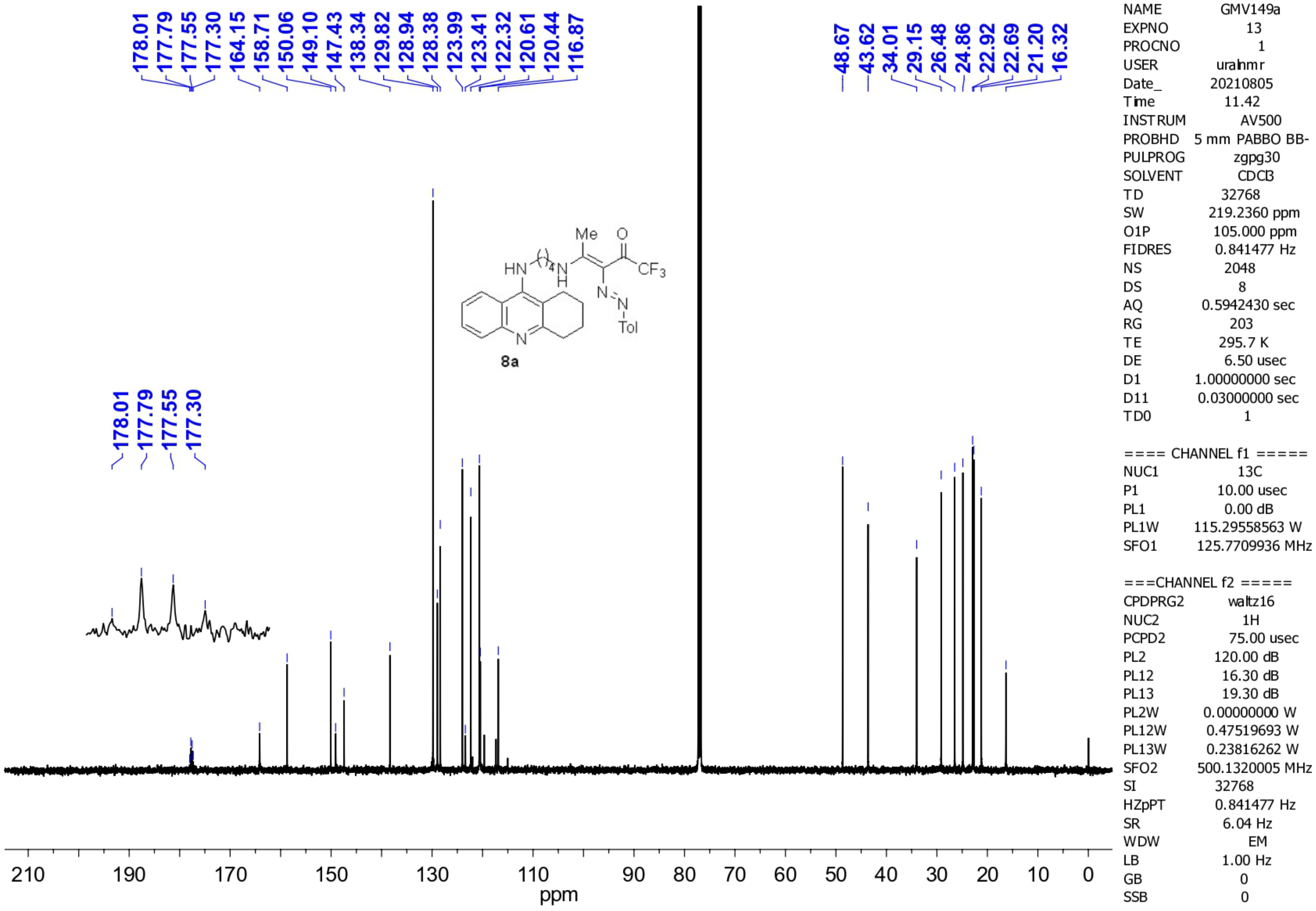

Figure S31. ^{13}C NMR spectrum of compound **8a**

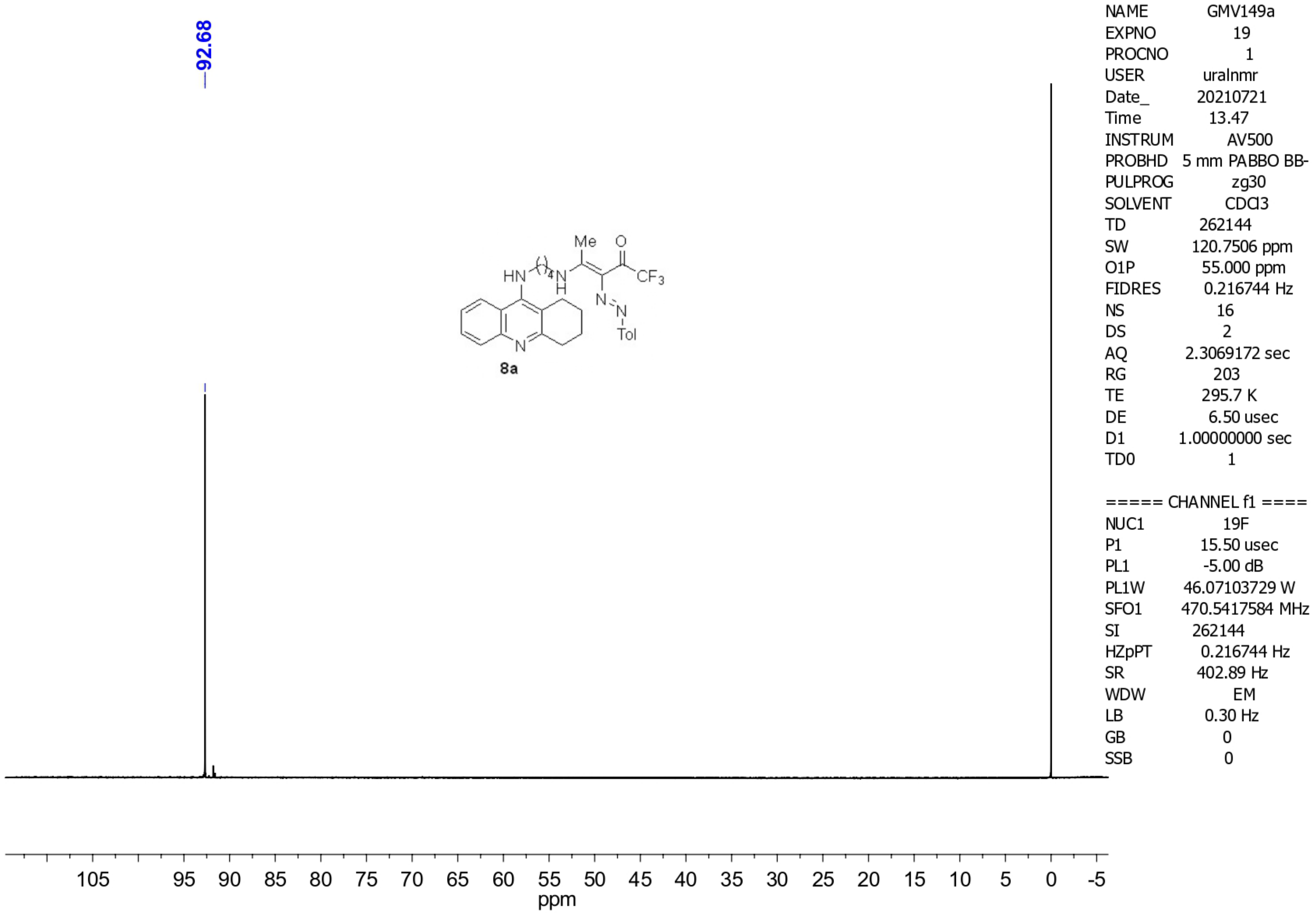

Figure S32. ^{19}F NMR spectrum of compound **8a**

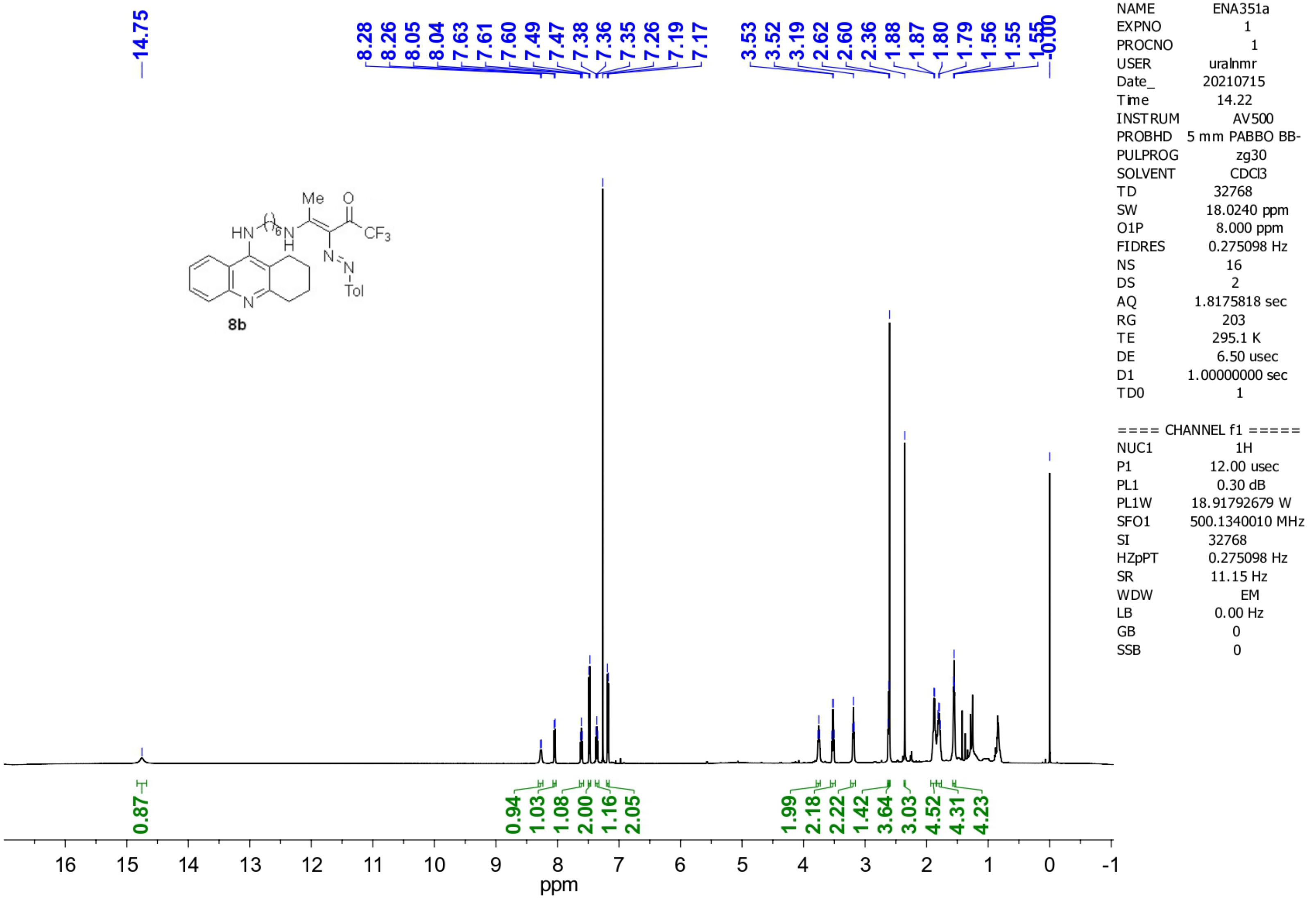

Figure S33. ^{1}H NMR spectrum of compound **8b**

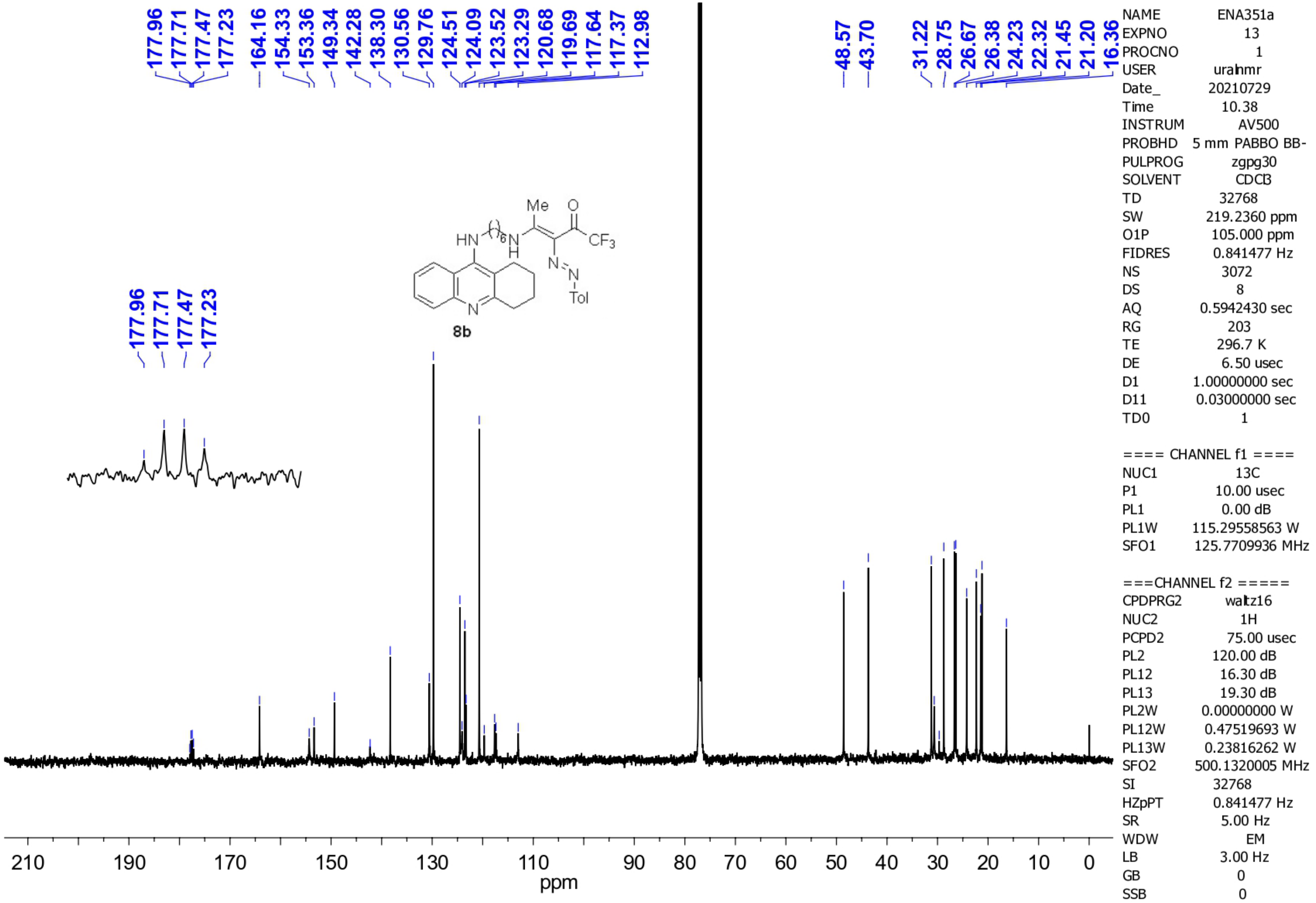

Figure S34. ^{13}C NMR spectrum of compound **8b**

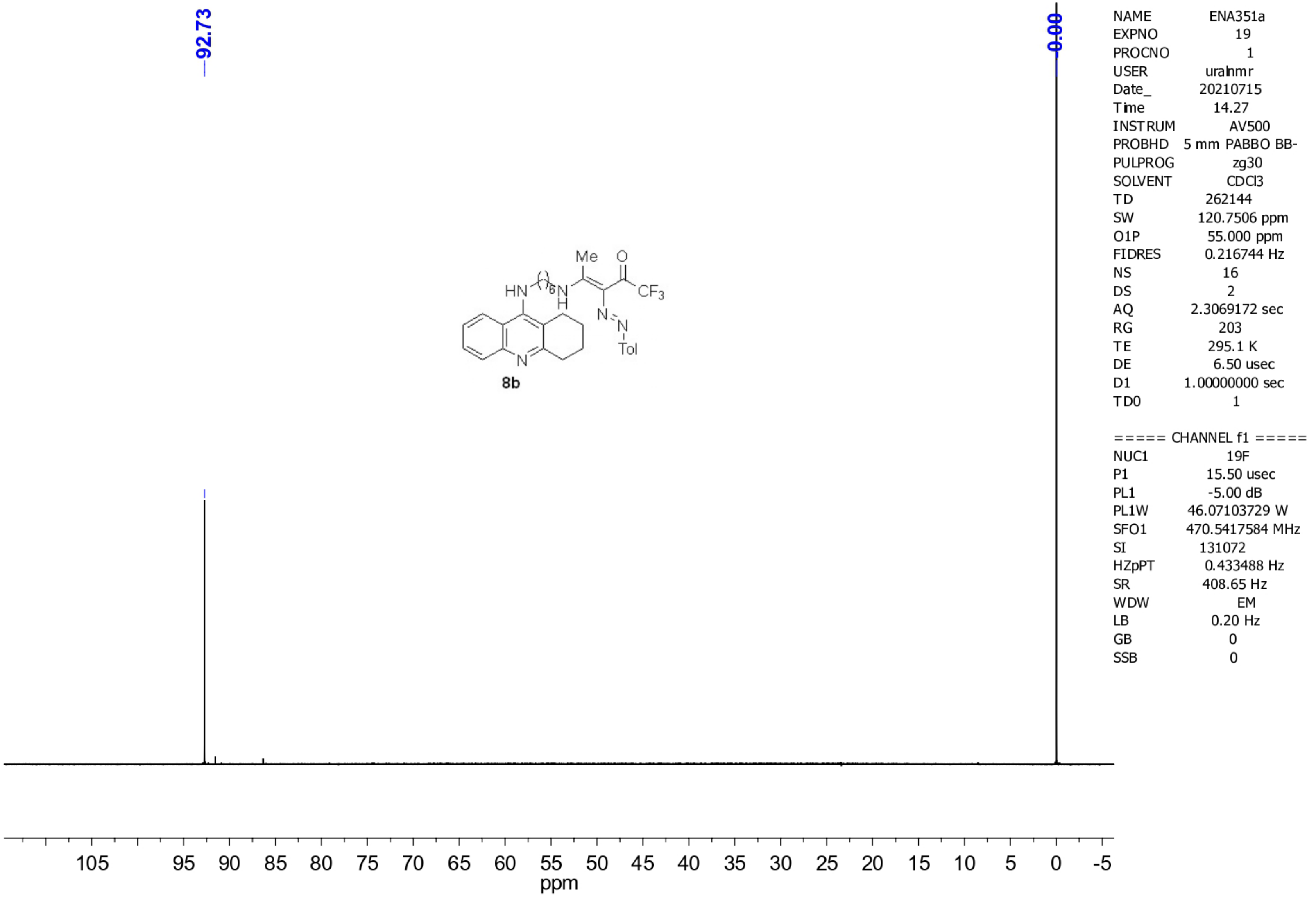

Figure S35. ^{19}F NMR spectrum of compound **8b**

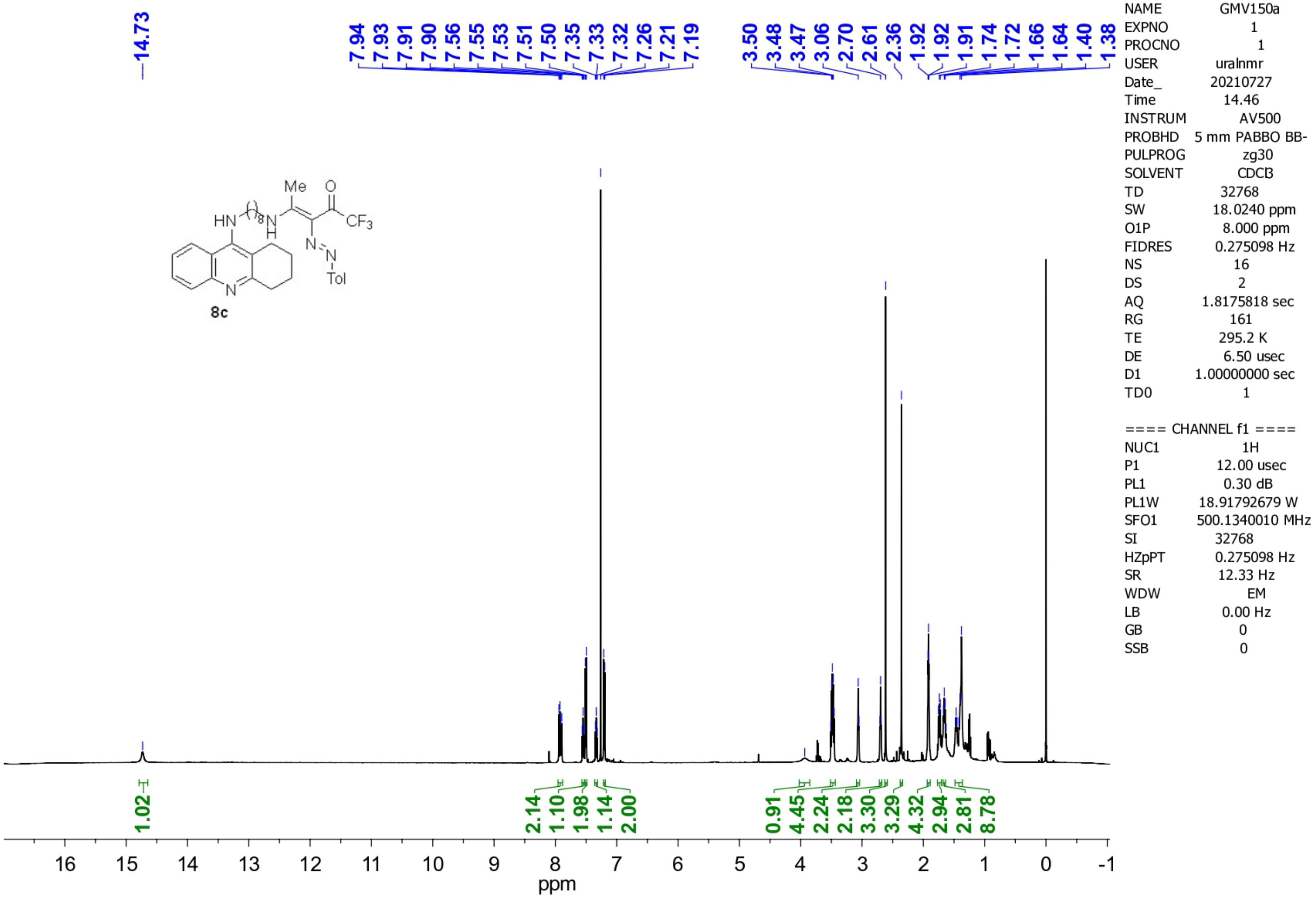

Figure S36. ^{1}H NMR spectrum of compound **8c**

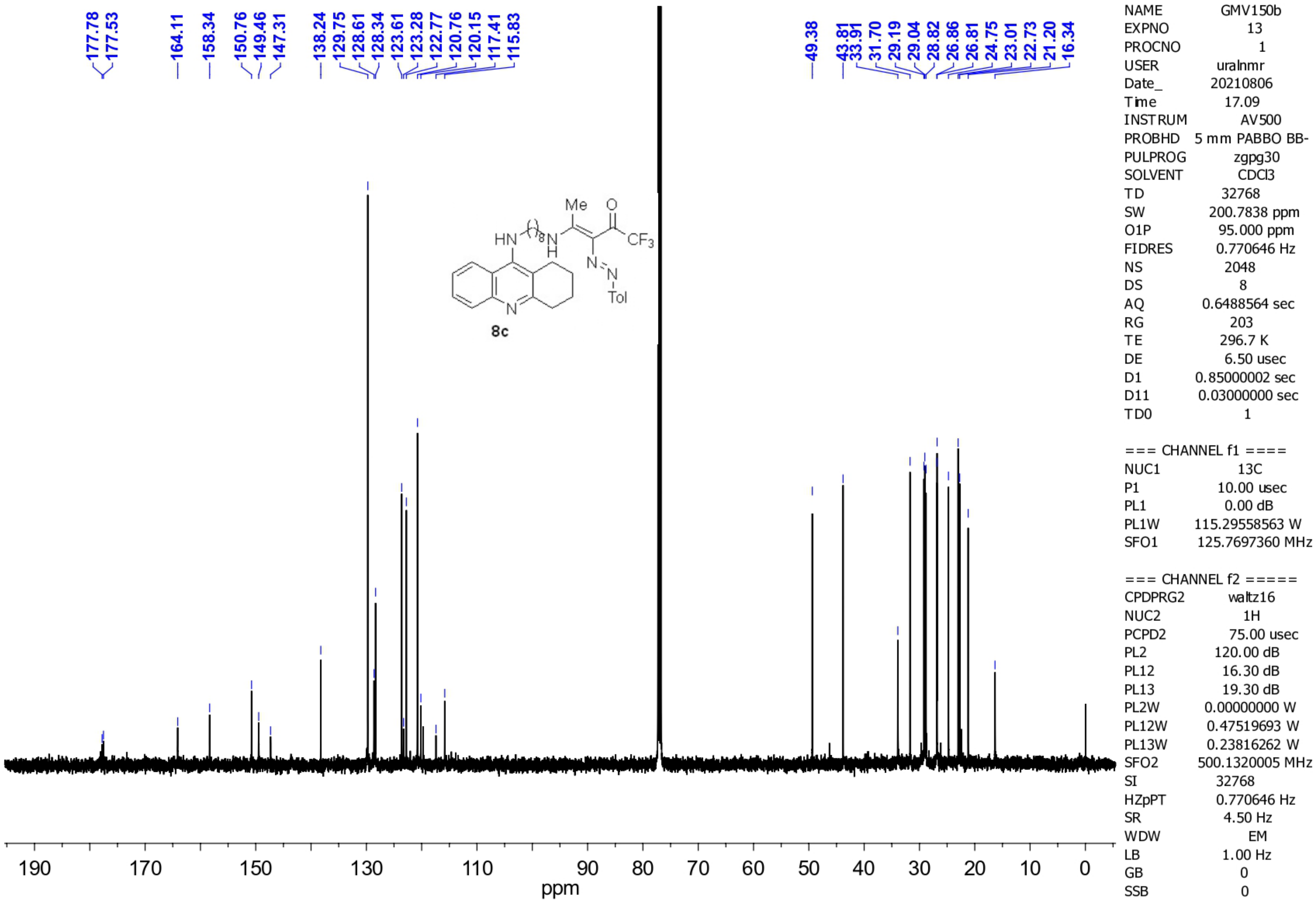

Figure S37. ^{13}C NMR spectrum of compound **8c**

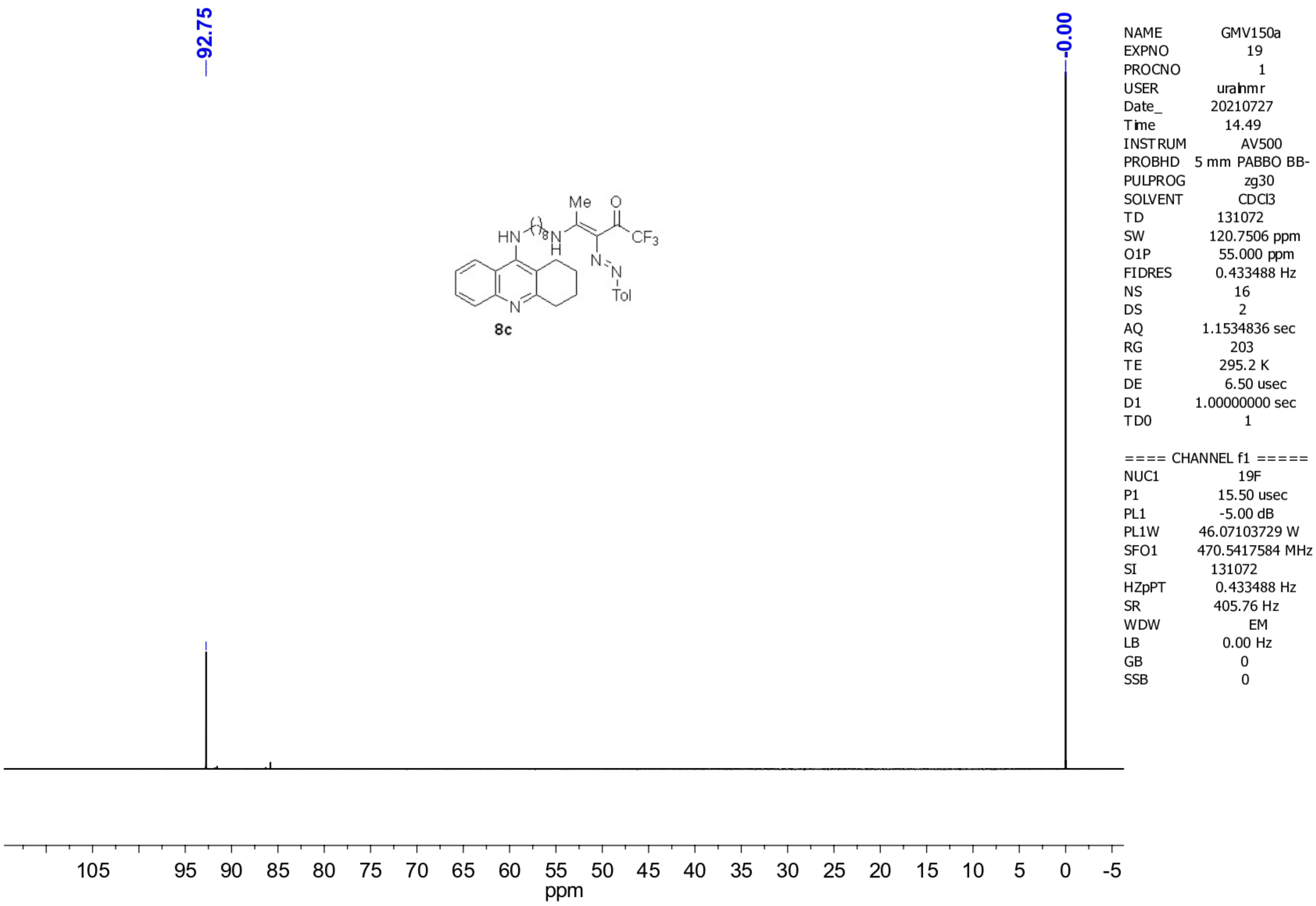

Figure S38. ^{19}F NMR spectrum of compound **8c**
